# Persistent sodium currents in neurons: potential mechanisms and pharmacological blockers

**DOI:** 10.1007/s00424-024-02980-7

**Published:** 2024-07-05

**Authors:** Peter Müller, Andreas Draguhn, Alexei V. Egorov

**Affiliations:** 1 Department Neurology and Epileptology, Hertie Institute for Clinical Brain Research, University of Tuebingen , Hoppe-Seyler-Straße 3, 72076 Tübingen, Germany; 2 Institute for Physiology and Pathophysiology, Medical Faculty, Heidelberg University, Im Neuenheimer Feld 326, 69120 Heidelberg, Germany

**Keywords:** Persistent sodium current, Slow inactivation, Epilepsy, Sodium channel blocker, Neuron

## Abstract

Persistent sodium current (I_NaP_) is an important activity-dependent regulator of neuronal excitability. It is involved in a variety of physiological and pathological processes, including pacemaking, prolongation of sensory potentials, neuronal injury, chronic pain and diseases such as epilepsy and amyotrophic lateral sclerosis. Despite its importance, neither the molecular basis nor the regulation of I_NaP_ are sufficiently understood. Of particular significance is a solid knowledge and widely accepted consensus about pharmacological tools for analysing the function of I_NaP_ and for developing new therapeutic strategies. However, the literature on I_NaP_ is heterogeneous, with varying definitions and methodologies used across studies. To address these issues, we provide a systematic review of the current state of knowledge on I_NaP_, with focus on mechanisms and effects of this current in the central nervous system. We provide an overview of the specificity and efficacy of the most widely used I_NaP_ blockers: amiodarone, cannabidiol, carbamazepine, cenobamate, eslicarbazepine, ethosuximide, gabapentin, GS967, lacosamide, lamotrigine, lidocaine, NBI-921352, oxcarbazepine, phenytoine, PRAX-562, propofol, ranolazine, riluzole, rufinamide, topiramate, valproaic acid and zonisamide. We conclude that there is strong variance in the pharmacological effects of these drugs, and in the available information. At present, GS967 and riluzole can be regarded *bona fide* I_NaP_ blockers, while phenytoin and lacosamide are blockers that only act on the slowly inactivating component of sodium currents.

## Introduction

**Fig. 1:**
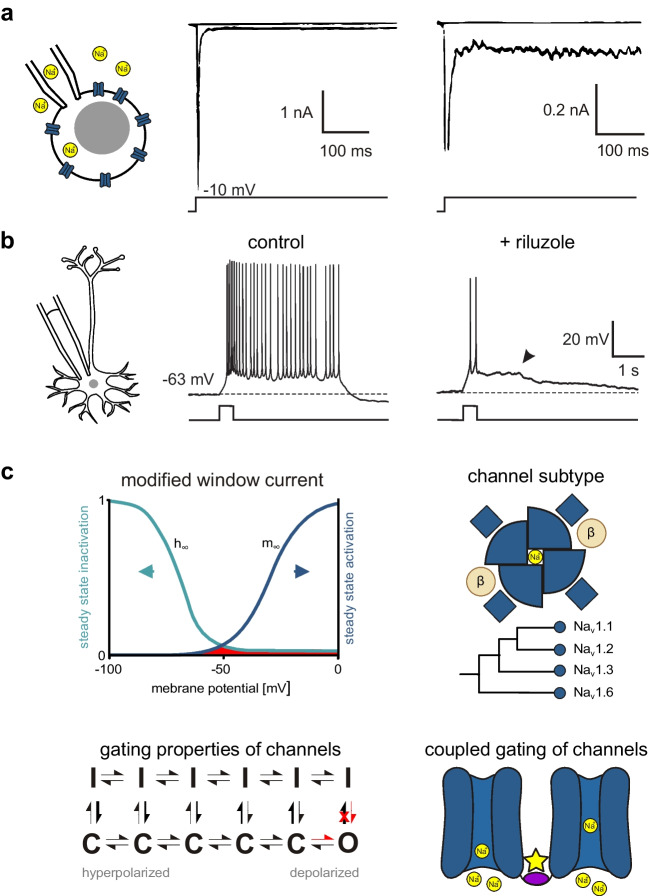
Physiological characterization of persistent sodium current. **a** Persistent sodium current (I_NaP_) is a small fraction of isolated voltage-dependent sodium current, typically measured with voltage clamp in transfected cells or dissociated neurons (left). I_NaP_ (right) is the persisting inward current component following a transient fast Na^+^-mediated inward current (middle). Adapted from French et al. [70] with permission. **b** An example for I_NaP_-dependent bursting behaviour recorded in current clamp mode. I_NaP_ contributs to neuronal bursting during a depolarizing current step as well as to the followed plateau potential (indicated by arrowhead). Right panel shows response of neuron to depolarizing current step after bath application of I_NaP_ blocker riluzole (10 µM). Adapted from Sheroziya et al. [197] with permission. **c** Schematic diagrams of potential mechanisms underlying I_Na__P_: the modified window currents hypothesis (top left) claims that I_NaP_ (red) emerges in the ‘window’ between activation (m_∞_) and inactivation (h_∞_) of sodium currents in the Hodgkin-Huxley-model, where h∞ approaches a small positive value as V → ∞ [162, 210]. Arrows indicate potential mechanisms of I_NaP_ block by shift of activation to the right or left shift inactivation. A second hypothesis focuses on gating properties of sodium channels (bottom left). Taddese and Bean [216] assume a preference for inactivated states depending on membrane potential (thick arrows). The ‘modal gating’ hypothesis assumes a (temporary) failure of inactivation that allows inactivated channels to open (red). Another hypothesis claims that I_NaP_ depends on different channel subunit isoforms (top right; scheme of an α subunit with complementary β subunits and a phylogenetic tree of neuronally expressed α subunits). Finally, the supramolecular gating hypothesis (bottom right) argues that interactions between single sodium channels alters their gating kinetics (indicated by star)

Voltage-gated sodium current is a fundamental component of excitable cells in all animals with active movement. It is mediated by sodium-selective cation channels, which appeared in evolution before the origin of nervous systems [137]. In mammals, the main (α) subunit of voltage-gated sodium channels (VGSC) consists of 4 identical motives, each of which containing 6 transmembrane segments, a pore-forming loop and a voltage sensor in the fourth membrane-spanning helix. A core feature of voltage-gated sodium currents is their fast inactivation following activation by membrane depolarization [91]. In most recordings, however, a small portion of the sodium current does not vanish within a few milliseconds (Fig. 1A). This persistent sodium current component (I_NaP_) has been defined as a ‘non-inactivating or slowly inactivating sodium current’ [49, 119]. Note that, although the name of the current contains the word ‘persistent’, slowly inactivating components are explicitly included. Thus, an unambiguous identification of I_NaP_ requires the demonstration of a non- or slowly inactivating component in VGSC-mediated currents.

I_NaP_ is thought to contribute to multiple cellular functions. It regulates the excitability of neurons [15, 69, 121, 130] and, specifically, of axons [160, 213], it amplifies excitatory and inhibitory postsynaptic potentials (EPSP/IPSP) [38, 72, 174, 211, 212, 234], and it contributes to pacemaking [27, 116, 120, 229], resonance [99, 233, 249], bursting behaviour in adult [17, 214] and immature neurons [196, 197, 201, 220], place cell tuning [95] and network oscillations [114]. An example for I_NaP_-dependent bursting behaviour in immature entorhinal cortex neuron is shown in Fig. 1B. I_NaP_ is up-regulated in several disorders, underlining its clinical importance and therapeutic potential. These pathophysiological situations include hypoxia [86, 94], demyelination [85, 225], neurogenic pain [126], paroxysmal extreme pain disorder [63], temporal lobe epilepsy [235], monogenetic epileptic syndromes [139, 206, 239], neurodegeneration [103, 168, 173, 178, 199], hemiplegic migraine [20, 40], and spasticity following traumatic brain or spinal cord injury [32, 136]. I_NaP_ has been described to increase with aging [140], and it is acutely modulated by protein kinase C [4, 14, 67], G-protein subunits [141, 143], acetylcholine [156, 248], dopamine [79, 80, 152] and endogenous polyamines [65, 183]. These modulations may be of importance for the effects of drugs used in neurological or psychiatric disorders, e.g. substances affecting cholinergic or dopaminergic transmission.

Pharmacological blockers of persistent sodium current allow assessing its function in physiological experiments on living animals, brain slices, or single cells (in the latter, the current component can also be eliminated by the biophysical approach of dynamic clamp [210]). More importantly, blockers of I_NaP_ with favourable safety profile may be efficient drugs in the different clinical conditions listed above. However, there is no established standard for the use and validation of I_NaP_ blockers in different laboratory preparations, experiments on living animals or clinical treatment of humans. Previous reviews have already established that there is a large range of putative I_NaP_ blockers [206, 239], in addition to the even wider range of global sodium channel blocking substances [133, 200]. We will provide an overview of present knowledge on their selectivity for I_NaP_, their potency, and their specific effects on different kinetic properties of sodium channels. The review shall provide an up-to-date basis for experimental and translational work on this important regulator of cellular excitability. It will also highlight some conceptual and semantic problems with the concept of ‘persistent sodium currents’, which are reflected in the heterogeneity of protocols used to study I_NaP_.

## I_NaP_ – Characteristics and Underlying Mechanisms

The mechanisms underlying persistent sodium currents are not completely understood and are, most likely, heterogeneous. Here, we will briefly review the dominant hypotheses about the structural or functional basis for I_NaP_ (Fig. 1C). One potential explanation for the occurrence of I_NaP_ results from the canonical kinetic model of voltage-activated sodium currents, as described in the original Hodgkin-Huxley model. The overlap of steady-state activation and inactivation curves creates a range of potentials where some Na^+^ channels are activated while inactivation is not complete. This forms a ‘window’ of potentials where some sodium current should be present at any time. Modulation of the voltage-dependence of activation or inactivation can alter the size of the window current and, hence, I_NaP_ (see, e.g. the discussion of carbamazepine below). However, French et al. [70] showed that the properties of I_NaP_ are not fully explained by the ‘window current’—for example, I_NaP_ conductance increases with more depolarized membrane potentials, while the window current should decrease. In addition, this ‘window current’ overestimates I_NaP_ in simulations [210]. The contradiction arises partly, because the original Hodgkin-Huxley model does consider activation and inactivation to be independent of each other and assumes complete inactivation with increasing depolarization. Taddese and Bean [216] proposed a modified version of the ‘window current’ in mammalian neurons. In this model, steady-state inactivation is dependent on steady-state activation [3, 10, 11, 29], resulting in a small remaining current component even at highly positive voltages. This modified window current (Fig. 1C top left) can account for the presence of I_NaP_ at depolarized potentials and has been implemented in computational models [162].

A second approach derives I_NaP_ from complicated gating schemes of sodium channels using more flexible Markov models (Fig. 1C bottom left). Early models, derived from measurements in squid axons, suggested two different open states [41, 46]. Later modifications of these models for mammalian cells assume only one single open state. Persistent opening does then result from one of two alternative mechanisms: i) a preference of more depolarized channels to enter inactivation even when closed [10, 38, 216]; ii) modal gating of sodium channels, which can enter a non-inactivating state with sustained, burst-like openings [5, 171]. It has to be noted that from a kinetic standpoint the Markov model of Taddese and Bean [216] and the modified window current are identical.

The third hypothesis for the mechanism underlying I_NaP_ is the existence of a separate channel subtype with the respective kinetic properties [57]. Indeed, there are nine different known α subunits of VGSC, opening the possibility that I_NaP_ is a property of one or several specific subunits. However, evidence from the last decades supports the idea that many different α subunits can produce I_NaP_, at least those with strong expression in the brain (for Na_v_1.1 see [6, 112]; for Na_v_1.2 see [43, 186]; for Na_v_1.3 see [60, 215]; and for Na_v_1.6 see [186, 232]); Fig. 1C top right). Amongst these subunits, Na_v_1.6 seems to be responsible for a major portion of I_NaP_ in the central nervous system (CNS) [186]. However, about half of the persistent sodium current remains after selective knockout of Na_v_1.6 in rat neocortical layer 5 pyramidal neurons, pointing towards the importance of further subunits [115]. Nevertheless, the strong contribution of Na_v_1.6 may be responsible for the well-known left shift of the activation curve when comparing I_NaP_ to the transient component of sodium current (I_NaT_) [49, 119]. This shift would result from the biophysical properties of Na_v_1.6, which activates at more hyperpolarized membrane potentials than Na^+^ currents mediated by the other subunits [96] (note that this left shift would increase the window current, see Fig. 1C, top left). This is supported by the right-shift of the activation curve for persistent sodium currents in hippocampal CA1 neurons of mice lacking functional Na_v_1.6 [182]. Conversely, though, selective knockout of Na_v_1.6 in cortical pyramidal neurons left the voltage-dependence of activation unchanged [115].

In dorsal root ganglion (DRG) neurons, Na_v_1.8 and Na_v_1.9 have been suggested to be responsible for I_NaP_ [119]. These subunits mediate a long-lasting, non-inactivating current component in transduction of sensory signals, which are particularly important for nociceptive stimuli [2]. A special feature of these subunits is their low sensitivity to the sodium channel blocker tetrodotoxin (TTX). Whereas Na_v_1.1-Na_v_1.4, Na_v_1.6 and Na_v_1.7 can be blocked by nanomolar concentrations of TTX, Na_v_1.8 and Na_v_1.9 require millimolar concentrations [1]. However, in measurements of I_NaP_, TTX-resistant components are frequently regarded as leak current and, hence, subtracted before analysis. This may lead to an underestimation of the role of Na_v_1.8 and Na_v_1.9. In any case, it is unlikely that Na_v_1.8 and Na_v_1.9 are responsible for I_NaP_ in cortical neurons, as single cell transcriptomics of human and mouse cortex show no expression of both *SCN10A* (Na_v_1.8) and *SCN11A* (Na_v_1.9) [90].

The major pore forming α-subunits of sodium channels are complemented by two auxiliary β subunits. There is evidence that the presence of the β4 subunit increases persistent sodium currents, while adding the β1 subunit neutralizes this effect [6]. Knock out of β1 can lead to a paradoxical effect of sodium channel blockers, which then enhance, rather than suppress, persistent sodium current [226].

Finally, recent evidence suggests the existence of coupled gating between different individual voltage-gated sodium channels [44, 101]. This supra-molecular cooperativity may also be involved in the generation of persistent sodium current (Fig. 1C bottom right) [185].

In summary, there is evidence for several different mechanisms underlying I_NaP_, including contributions by specific molecular subtypes of α or auxiliary subunits and effects of gating kinetics. None of the explanations seems to account for all observations, and they are not mutually exclusive, suggesting convergence of several mechanisms to the generation of persistent sodium currents in many excitable cells.

## Electrophysiological Isolation of I_NaP_

**Fig. 2:**
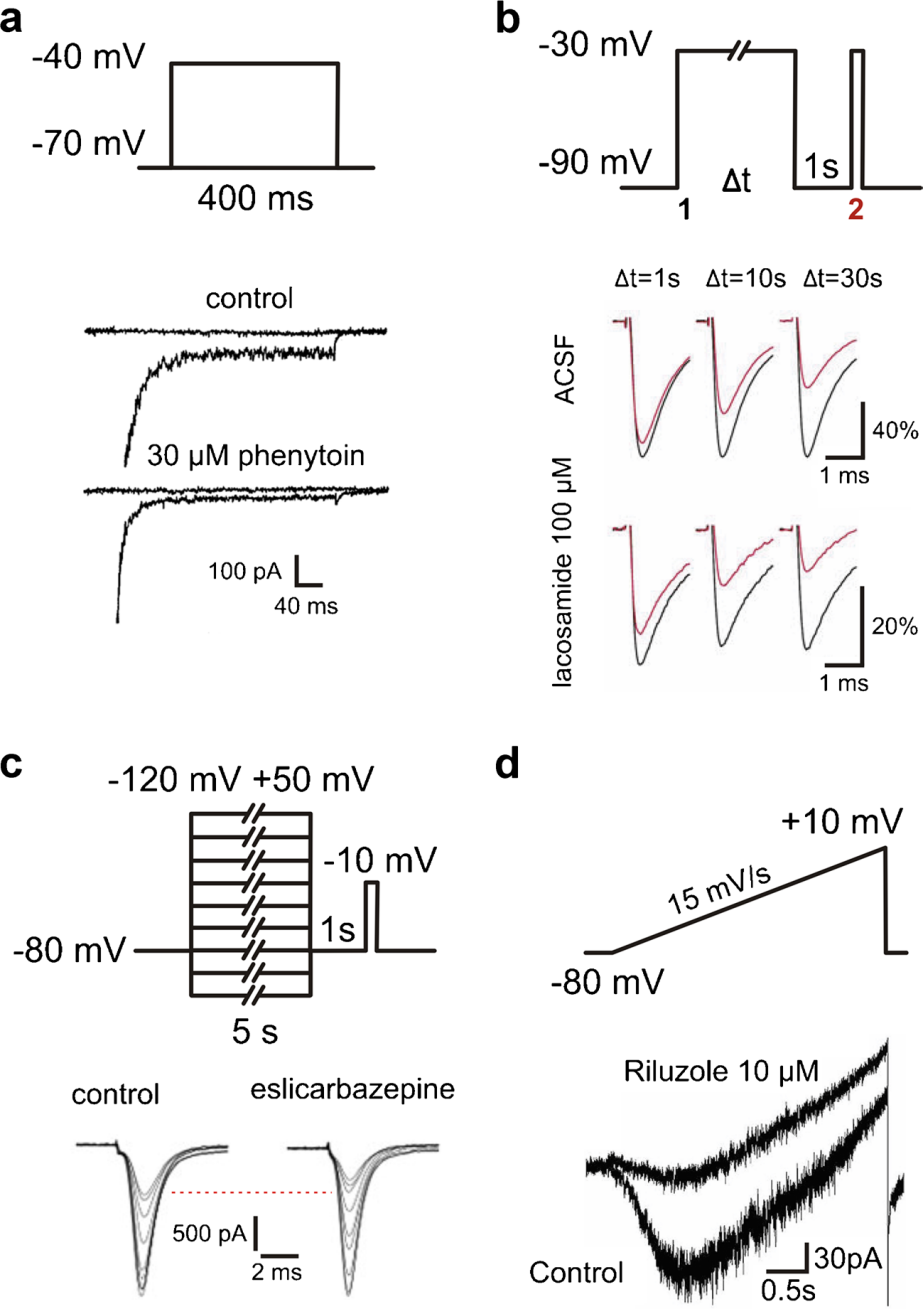
Different voltage clamp protocols for persistent sodium currents and exemplary drug effects. **a** Voltage step protocol (top) with exemplary current traces showing the effect of 30 µM phenytoin (bottom; adapted with permission from Chao and Alzheimer [42]). **b** Voltage step protocol for entry into slow inactivation with pulses of variable durations (top) with exemplary current traces showing the effect of 100 µM lacosamide (bottom; adapted with permission from Holtkamp et al. [92]). **c** Voltage step protocol for slow steady state inactivation (top) with exemplary current traces showing the effect of 250 µM eslicarbazepine (bottom; adapted with permission from Hebeisen et al. [87]). **d** Voltage ramp protocol (top) with current traces showing the effect of 10 µM riluzole (bottom; adapted with permission from Nakamura et al. [163])

Different voltage clamp protocols are used to isolate the persistent sodium current components in cells or isolated membranes. One frequently used protocol focusses on the early sodium current component, using depolarizing voltage steps of 50—500 ms duration (Fig. 2A). The inward current that persists at the end of this step is then defined as persistent component (Fig. 1A and 2A). Historically, this protocol did underly the first description of ‘late sodium current’ in frog axons [57]. This brief ‘step pulse’ method does, however, not exclude that the apparently persistent component inactivates with a slower time course, which is not visible within the time window of the test pulse.

In addition to the well-known fast inactivation of I_NaT_ with a time constant of < 10 ms, there are intermediate inactivation with a time constant of ~ 100 ms [71] and slow inactivation with a time constant ≥ 1 s [184]. It has to be noted that intermediate inactivation is not generally embraced by the literature and many inactivation protocols with pulse lengths greater than 100 ms likely study both fast and intermediate inactivation [71]. In the following, we will use the terms fast, intermediate and slow inactivation for the three kinetic components described here. In order to test for these additional types of inactivation, especially slow inactivation, long current pulses and repetitive activation steps have been used.

A simple option for testing slow inactivation employs very long stimuli to test whether activation of sodium current is impaired after them in comparison to before. A typical protocol for this purpose consists of three steps (Fig. 2B): First, a long (1 – 30 s) depolarizing pulse (e.g. -30 mV) from hyperpolarized potentials, then a brief (0.5–1 s) recovery pulse to hyperpolarized potentials and finally a short (15 ms) test pulse to a depolarized potential (e.g.—30 mV; see [92, 166, 195]). In theory, the recovery pulse allows fast, but not slow, inactivated channels to recover from inactivation. The test pulse then determines this fraction of channels, such that the slowly inactivated fraction can be calculated (Fig. 2B). We will call this protocol entry into slow inactivation.

Alternatively, the voltage dependence of slow inactivation was examined using a protocol in which long voltage pulses varying between -120 and + 50 mV (1–10 s duration) were followed by a recovery pulse to a hyperpolarized potential (duration 0.5–1 s) and a test pulse (10 ms duration) to depolarized potential (e.g. -10 mV). This protocol allows to analyse the voltage-dependent amount of inactivated channels and offers a fuller picture of the effects of a drug (Fig. 2C). We will call this protocol slow steady state inactivation.

A problem with both approaches lies in the theoretical assumption that there are only fast and slow inactivation: The length of the recovery pulse is inconsistent in the literature and after longer recovery pulses, channels might have already recovered from intermediate inactivation.

If sodium channels were to undergo fast, intermediate and slow inactivation, at some point there would eventually be no current and the term ‘persistent’ sodium current would obviously be misleading [45]. However, for both of the aforementioned examples, inactivation never fully completes during the depolarizing pulses. Whether or whether not there is then truly persistent, non-inactivating sodium current, remains an open question.

Another more broadly applied approach employs slow voltage ramps, typically with velocities of 10–70 mV/s ranging from -80—+ 10 mV (Fig. 2D). At these slow depolarization velocities, the transient component I_NaT_ is thought to inactivate, such that the remaining component should isolate I_NaP_. However, depolarizing a cell at such slow speed may induce some slow inactivation, leading to a potential underestimation of the remaining I_NaP_. This pitfall explains the hysteresis of slow current components (I_NaP_) between ascending and descending voltage ramps, a well-known hallmark of persistent sodium current [25]. Different ramp speeds may lead to different resulting currents, including potential distortions of the ‘true’ I_NaP_. For example, Fleidervish and Gutnick [64] found that a high depolarization speed of 233 mV/s induced action currents (a Na_V_-generated escape phenomenon in voltage clamp recordings) in cortical pyramidal neurons, while slower ramps of 70 mV/s did not. The velocity of voltage ramps may also affect the apparent effectiveness of I_NaP_ blockers, if these have differential effects on different inactivation components. For instance, slower ramp speeds result in larger TTX-susceptible current components and fast ramp speeds underestimate the effect of phenytoin (see Table 1, [45]). Frequently, ramps can be employed in neurons with complex morphology to reduce the space-clamp error [12, 162]. In such experiments, the difference between ramp-induced currents in the absence and presence of TTX is measured and taken as a proxy for I_NaP_, as long as TTX resistant sodium channels are absent [163]. The validation with TTX is important, as leak, potassium and calcium currents are also being evoked with this protocol. Interpretation of the resulting traces is complex, as exemplified by a study characterizing fluoxetine as an apparent I_NaP_ blocker, based on a ramp protocol that actually displays a clear potassium current block [100]. Altogether, voltage ramp measurements are less precise than step-protocols, but they are easier to implement in complex, extended cells like naturally differentiated neurons in ex vivo brain slices. Slow command voltage changes reduce the space-clamp error and help to avoid voltage-clamp escape phenomena like ‘action currents’. Arguably, they are a somewhat more physiological command than a sudden voltage step.

Few papers have studied the effect of slow inactivation protocols on subsequent ramps. While these protocols might be one of the best ways of assessing persistent sodium current, they are very difficult to record and therefore only seldomly employed [45, 123, 242].

## Pharmacology of I_NaP_

**Fig. 3:**
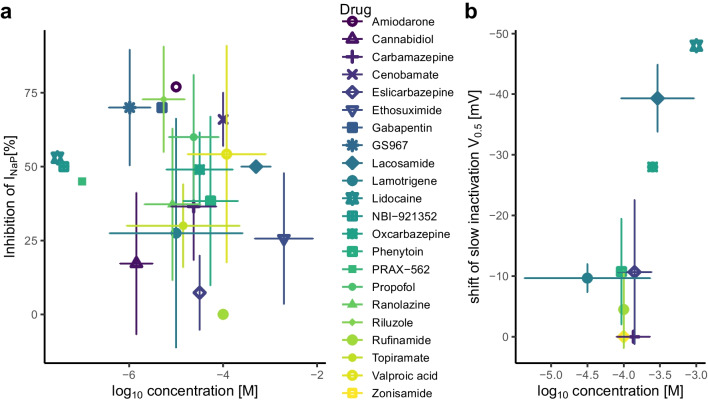
Summary of drug effects with respect to protocol. Each study value listed in the table was considered as a separate datum, values plotted are mean ± SD. **a** Inhibition of persistent sodium current (I_NaP_) assessed by step pulse or ramp protocols plotted vs drug concentration (values taken from [8, 16, 18, 20, 35, 42, 45, 53, 55, 76, 86, 114, 122, 125, 129, 130, 135, 149, 150, 163, 165, 170, 173, 175, 205, 215, 217, 221, 226–228, 240, 244, 246, 251, 253]). **b** Shift of the half point of voltage-dependent slow steady state inactivation curve plotted vs drug concentrations (values taken from [21, 78, 93, 107, 110, 124, 151, 166, 195, 226, 253])

Our systematic literature search identified 2586 PubMed results for persistent, slowly or late inactivating sodium currents in combination with blocking, reducing or inhibiting drug actions. Our search term was ‘(((persistent) OR (slow inactivation) OR (late)) AND (sodium current)) AND ((block) OR (inhibit) OR (reduce))’. Papers were first screened for pharmacological agents with potential clinical applications, i.e. excluding endogenous substances (e.g. acetylcholine), toxins (e.g. saxitoxin), chemicals without present or planned clinical use (e.g. insecticides or dyes) and intracellular agents (e.g. QX-314). After identification of potential blockers, a second literature search was conducted with ‘(((persistent) OR (slow inactivation) OR (late)) AND (sodium current)) AND *substance name*’) for each substance. Papers were considered eligible when appropriate voltage clamp protocols for either non-inactivating or slowly inactivating sodium currents were applied to CNS neurons or to cells expressing Na_v_1.1, Na_v_1.2, Na_v_1.3 or Na_v_1.6. We will now discuss the actions of the identified substances used in I_NaP_-related research as listed in Table 1 (see also Fig. 3 for an overview). We will consider their specificity, affinity and different mechanisms of action. Where applicable, clinically relevant information such as blood concentrations and blood–brain-barrier interactions (a major problem for clinical translation) will be included. Concentrations are given as IC_50_ or EC_50_ values, respectively.

**Table 1:** List of drug effects sorted by studies. Studies included had to be performed on neuronal sodium channels or neurons. Furthermore, they needed to employ either a step protocol or ramp protocol for measuring I_NaP_ or a protocol for slow inactivation. Only voltage clamp protocols were eligible to be included in this list. Whenever possible EC_50_ or IC_50_ values are given. HP: holding potential, n.s.: not significant, V_0.5_:midpoint voltage, IC_50_: half-maximal inhibitory concentration, EC_50_: half-maximal effect concentration, EC_max_: maximal effect size

Drug	Preparation	Protocol for I_NaP_	Effect on I_NaP_	Effect on I_NaT_	Study
**Amiodarone**					
10 µM	rat neocortical neurons	60 mV/s ramp	-77% peak amplitude	not assessed	Spadoni et al. [205]
**Cannabidiol**					
1 µM	HEK293 cells expressing human Na_v_1.1	step pulse 180 ms	n.s. effect on amplitude	n.s. shift of midpoint voltage (V_0.5_) of steady state inactivation 500 ms	Patel et al. [170]
	HEK293 cells expressing human Na_v_1.6	step pulse 180 ms	n.s. effect on amplitude	n.s. shift of V_0.5_ of steady state inactivation 500 ms	
	rat striatal neurons	step pulse 180 ms	-36% peak amplitude	4 mV left shift of steady state inactivation 500 ms	
3 µM	HEK cells expressing a human Na_v_1.6 mutation	step pulse 100 ms	IC_50_ 6 µM	-50% amplitude at holding potential (HP) -60 mV (IC_50_ 3 µM)	Ghovanloo et al. [76]
1 µM	HEK cells expressing human Na_v_1.2	step pulse 50 ms	n.s. effect on peak amplitude	-22% average current density; n.s. effect on peak current density n.s. shift of V_0.5_ of steady state inactivation 500 ms	Mason and Cummins [150]
**Carbamazepine **					
10 µM	HEK293 cells expressing human Na_v_1.3	step pulse 100 ms	-20% amplitude (EC_50_ 16 µM E_max_ -46%)	3 mV left V_0.5_ of steady state inactivation 500 ms (EC_50_ 14 µM E_max_ 8 mV)	Sun et al. [215]
100 µM	HEK293 cells expressing Na_v_1.3	entry into slow inactivation 10 s	IC_50_ 406 µM	n.s. effect on amplitude at holding potential (HP) -120 mV (IC_50_ 2464 µM)	Sheets et al. [195]
1 mM		slow steady state inactivation 10 s	n.s. effect on V_0.5_	19 mV V_0.5_ left shift of steady state inactivation 500 ms	
100 µM	Scn1b wildtype mouse dissociated dentate gyrus neurons	50 mV/s ramp	-48% peak amplitude 8 mV V_0.5_ left shift	-32% peak amplitude (HP -100) 8 mV V_0.5_ left shift of activation	Uebachs et al. [226]
100 µM	N1E-115 neuroblastoma cells	slow steady state inactivation 10 s	n.s. effect on V_0.5_	not assessed	Niespodziany et al. [166]
250 μM	N1E-115 neuroblastoma cells	entry into slow inactivation 30 s slow steady state inactivation 5 s	n.s. effect on amplitude n.s. effect on V_0.5_	n.s. V_0.5_ left shift of activation -24% amplitude (HP -80 mV; HP -100 mV IC_50_ 822 µM; HP -80 mV IC_50_ 399 µM; HP -60 mV IC_50_ 109 µM) 7 mV V_0.5_ left shift of steady state inactivation 500 ms	Hebeisen et al. [87]
100 µM	HEK-293 cells expressing hNa_v_1.6 perfused with ATX-II	step pulse 200 ms	-56% peak amplitude (IC_50_ 77 µM)	-13.8% peak amplitude (IC_50_ 2307 µM, HP -120 mV)	Kahlig et al. [111]
		entry into slow inactivation 8 s	-63% peak amplitude (IC_50_ 44 µM)		
30 µM	Neuro-2a cells	500 mV/s ramp	-22% peak amplitude	-13% peak amplitude (HP -100 mV, IC_50_ 56 µM)	Wu et al. [245]
**Cenobamate**					
100 µM	rat CA3 pyramidal neurons	step pulse 150 ms	-74% amplitude (IC_50_ 53 µM)	-5% peak amplitude (HP -80 mV; IC_50_ > 500 µM)	Nakamura et al. [163]
		15 mV/s ramp	-68% peak amplitude (IC_50_ 53 µM)	6 mV V_0.5_ left shift of steady state inactivation 500 ms (EC_50_ 48 µM EC_max_ 10 mV)	
		entry into slow inactivation 8 s	-68% peak amplitude		
100 µM	HEK-293 cells expressing hNa_v_1.6 perfused with ATX-II	step pulse 200 ms entry into slow inactivation 8 s	-56% peak amplitude (IC_50_ 72 µM) IC_50_ 67 µM	amplitude: IC_50_ 1719 µM, HP -120 mV	Kahlig et al. [111]
**Eslicarbazepine**					
300 µM	Scn1b wildtype mouse dissociated dentate gyrus neurons	50 mV/s ramp	-22% peak amplitude 2 mV left shift of activation	not assessed	Doeser et al. [55]
250 µM	N1E-115 neuroblastoma cells	entry into slow inactivation 30 s slow steady state inactivation 5 s	-41% amplitude 31 mV V_0.5_ left shift	n.s. V_0.5_ shift of activation -6% amplitude (HP -80 mV; HP -100 mV IC_50_ 15,744 µM; HP -80 mV IC_50_ 3106 µM; HP -60 mV IC_50_ 562 µM) n.s. V_0.5_ shift of steady state inactivation 500 ms	Hebeisen et al. [87]
300 µM	rat dissociated dentate granule neurons	entry into slow inactivation 1 s	-4% peak amplitude	-23% peak amplitude (HP -90 mV)	Holtkamp et al. [93]
		entry into slow inactivation 10 s	-8% peak amplitude		
		entry into slow inactivation 30 s	-6% peak amplitude		
100 µM	CHO/HEK with Na_v_1.1	slow steady state inactivation 10 s	n.s. effect on V_0.5_	not assessed	
		entry into slow inactivation 10 s	n.s. effect on peak amplitude		
	CHO/HEK with Na_v_1.2	slow steady state inactivation 10 s	10 mV V_0.5_ left shift	not assessed	
		entry into slow inactivation 10 s	-48% peak amplitude		
	CHO/HEK with Na_v_1.3	slow steady state inactivation 10 s	n.s. effect on V_0.5_	not assessed	
		entry into slow inactivation 10 s	n.s. effect on peak amplitude		
	CHO/HEK with Na_v_1.6	slow steady state inactivation 10 s	6 mV V_0.5_ left shift	not assessed	
		entry into slow inactivation 10 s	-54% peak amplitude		
300 µM	ND7/23 cells with Na_v_1.6	slow steady state inactivation 30 s	17 mV V_0.5_ left shift	3 mV V_0.5_ left shift of fast	Bayraktar et al. [21]
		step pulse 100 ms	n.s. effect on amplitude	steady state inactivation 100 ms	
		175 mV/S ramp	n.s. effect on amplitude	n.s. V_0.5_ shift of activation	
**Ethosuximide**					
750 µM	rat thalamocortical neurons	200 mV/s ramp	-40% peak amplitude	n.s. effect on amplitude (HP -70 mV)	Leresche et al. [135]
1 mM	rat CA1 pyramidal neurons	step pulse 250 ms	small n.s. decrease in amplitude	not assessed	Niespodziany et al. [165]
10 mM	rat TC neuron	75 mV/s ramp	-37% peak amplitude	not assessed	Broicher et al. [33]
**Gabapentin**					
5 µM	rat DRG neurons	20 mV/s ramp	-70% peak amplitude	n.s. effect on amplitude (HP -120 mV)	Yang et al. [250]
**GS967**					
1 µM	mouse hippocampal pyramidal neurons with a Na_v_1.2 mutation	step pulse 200 ms	-92% amplitude	not assessed	Anderson et al. [8]
	tsA201 cells transfected with a Na_v_1.2 mutation	step pulse 200 ms	IC_50_ 0.4 µM	IC_50_ 19 µM for amplitude (HP -90 mV)	
200 nM	mouse hippocampal pyramidal neurons with a Na_v_1.6 mutation	step pulse 200 ms	-93% amplitude	n.s. effect on amplitude (HP -120 mV)	Baker et al. [18]
5 µM	Xenopus oozytes transfected with Na_v_1.1 mutations	step pulse 70 ms	-70% amplitude	-10% peak amplitude (HP -90 mV)	Barbieri et al. [20]
1 µM	ND7/23 cells transfected with a Na_v_1.6 mutation	step pulse 100 ms slow steady state inactivation 1 s with 5 ms recovery pulse	-83% amplitude 15 mV left shift	n.s. effect on amplitude (HP -120 mV)	Bunton-Stasyshyn et al. [35]
1 µM	ND7/23 cells transfected with Na_v_1.6 wildtype	step pulse 80 ms	no I_NaP_ found	n.s. effect on amplitude (HP -120 mV) 17 mV left shift of steady state inactivation 500 ms	Wengert et al. [240]
	mouse subiculum pyramidal neurons with Na_v_1.6 wildtype	65 mV/s ramp	-49% peak amplitude	not assessed	
1 µM	HEK cells expressing wild type human Na_v_1.2 channels	step pulse 50 ms	-45% current density	-35% peak current density (HP -100 mV)	Mason and Cummins [150]
3 µM	mouse hippocampal fast-spiking neuron	25 mV/s ramp	-78% area under the curve	not assessed	Auffenberg et al. [16]
**Lacosamide**					
100 µM	mouse N1E-115 neuroblastoma cells	entry into slow inactivation 30 s	-43% peak amplitude	-28% amplitude at HP -100 mV -29% amplitude at HP -60 mV n.s. V_0.5_ shift of steady state inactivation 500 ms	Errington et al. [59]
100 µM	HEK293 cells expressing Na_v_1.3	entry into slow inactivation 10 s	IC_50_ 415 µM	n.s. effect on amplitude at HP -120 mV (IC_50_ 51 mM) -25% peak amplitude at HP -80 mV	Sheets et al. [195]
1 mM		slow steady state inactivation 10 s	42 mV V_0.5_ left shift	n.s. V_0.5_ shift of steady state inactivation 500 ms	
300 µM	mouse dissociated hippocampal neurons	50 mV/s ramp	-50% peak amplitude	not assessed	Uebachs et al. [227]
100 µM	N1E-115 neuroblastoma cells	slow steady state inactivation 10 s	33 mV V_0.5_ left shift	not assessed	Niespodziany et al. [166]
250 µM	N1E-115 neuroblastoma cells	entry into slow inactivation 10 s steady state inactivation 5 s	-14% peak amplitude 43 mV V_0.5_ left shift	n.s. V_0.5_ left shift of activation -20% peak amplitude (HP -80 mV) n.s. V_0.5_ shift of steady state inactivation 500 ms	Hebeisen et al. [87]
100 µM	rat dissociated hippocampal granule cells	entry into slow inactivation 1 s entry into slow inactivation 10 s entry into slow inactivation 30 s	-8% peak amplitude -14% peak amplitude n.s. effect on peak amplitude	4 mV V_0.5_ left shift of fast steady state inactivation	Holtkamp et al. [92]
100 µM	HEK-293 cells expressing hNa_v_1.6 perfused with ATX-II	step pulse 200 ms entry into slow inactivation 8 s	-9.6% amplitude (IC_50_ 832 µM) IC_50_ 269 µM	not assessed	Kahlig et al. [111]
**Lamotrigene**					
100 µM	rat dissociated hippocampal neurone	slow steady state inactivation 9 s entry into slow inactivation 6 s	11 mV V_0.5_ left shift -69% peak amplitude	-7% amplitude at HP -90 mV (IC_50_ 1490 µM) -98% amplitude at HP -60 mV (IC_50_ 7 µM)	Kuo and Lu [124]
1 µM	rat neocortical pyramidal neurons	60 mV/s ramp	n.s. effect on amplitude	n.s. effect on amplitude (HP -65 mV)	Spadoni et al. [205]
100 µM	rat neocortical layer 5 pyramidal neurons	10 mV/s ramp	not quantified but similar to 100 µM phenytoin	responses ‘still inducible’	Berger and Lüscher [26]
30 µM	HEK cells expressing hNa_v_1.2	slow steady state inactivation 30 s	11 mV V_0.5_ left shift	n.s. effect on amplitude at HP -100 mV (IC_50_ 2.6 mM) -33% peak amplitude at HP -60 mV (IC_50_ 172 µM) n.s. shift of activation n.s. shift of fast steady state inactivation 10 ms	Jones et al. [107]
100 µM	N1E-115 neuroblastoma cells	slow steady state inactivation 10 s	7 mV V_0.5_ right shift	not assessed	Niespodziany et al. [166]
100 µM	HEK-293 cells expressing hNa_v_1.6 perfused with ATX-II	step pulse 200 ms	-55.4% amplitude (IC_50_ 78 µM)	-13.8% peak amplitude (IC_50_ 1249 µM, HP -120 mV)	Kahlig et al. [111]
		entry into slow inactivation 8 s	-72.8% amplitude (IC_50_ 39 µM)		
**Lidocaine**					
30 nM	rat dissociated CA1 pyramidal neurons	step pulse 500 ms	-53% amplitude	n.s. effect on amplitude (HP -100 mV)	Hammarstrom and Gage [86]
10 µM	rat DRG neurons	15 mV/s ramp	‘obvious’ effect, not quantified	‘little’ effect, not quantified	Dong et al. [56]
100 µM	HEK293 cells expressing Na_v_1.3	entry into slow inactivation 10 s	IC_50_ 284 µM	-6% amplitude at HP -120 mV (IC_50_ 1462 µM)	Sheets et al. [195]
1 mM		slow steady state inactivation 10 s	48 mV V_0.5_ left shift	20 mV V_0.5_ left shift of steady state inactivation 500 ms	
**NBI-921352**					
41 nM	HEK293 cells expressing a hNa_v_1.6 mutation	step pulse 20 ms	-50% amplitude	IC_50_ 33 µM, HP -120 mV IC50 53 nM, HP -62 mV	Johnson et al. [106]
**Oxcarbazepine**					
10 µM	NG108-15 cells	100 mV/s ramp	-40% peak amplitude		Huang et al. [97]
3 µM				-9 mV shift of fast steady state inactivation 30 ms -61% amplitude (HP -80 mV)	
250 µM	N1E-115 neuroblastoma cells	entry into slow inactivation 30 s slow steady state inactivation 5 s	n.s. effect on amplitude 28 mV V_0.5_ left shift	-24% amplitude (HP -80 mV HP -100 mV IC_50_ 2000 µM; HP -80 mV IC_50_ 805 µM; HP -60 mV IC_50_ 173 µM) 17 mV left V_0.5_ shift of steady state inactivation 500 ms	Hebeisen et al. [87]
100 µM	HEK-293 cells expressing hNa_v_1.6 perfused with ATX-II	step pulse 200 ms	-57.8% amplitude (IC_50_ 123 µM)	IC_50_ 1035 µM, HP -120 mV	Kahlig et al. [111]
		entry into slow inactivation 8 s	IC_50_ 42 µM		
**Phenytoin**					
75 µM	N1E-115	entry into slow	20 mV V_0.5_ left shift		Matsuki et al. [151]
100 µM	neuroblastoma cells	inactivation 60 s		-6% amplitude at HP -100 mV -42% amplitude at HP -80 mV	
100 µM	rat CA1 neurons	slow steady state inactivation 16 s	15 mV V_0.5_ left shift	-10% amplitude at HP -90 mV (IC_50_ 600 µM) -95% amplitude at HP -50 mV (IC_50_ 7 µM) n.s. V_0.5_ shift of fast steady state inactivation 100 ms	Kuo and Bean [123]
		entry into slow inactivation 12 s	-85% peak amplitude		
34 µM	rat neocortical and neostriatal neurons	step pulse 400 ms	-50% amplitude (EC_50_ 34 µM)	not assessed	Chao and Alzheimer [42]
		50 mV/s ramp	no V_0.5_ shift		
60 µM	hippocampal neurons in culture	single channel recordings in outside-out patches	-77% amplitude of late (50 – 100 ms) currents	-60% amplitude (HP -100 mV)	Segal and Douglas [192]
75 µM	rat neocortical layer 5 pyramidal neurons	7.3 mV/s ramp	-40% peak amplitude (EC_50_ 78 µM, EC_max_ -90%)	not assessed	Lampl et al. [130]
1 µM	rat neocortical pyramidal neurons	60 mV/s ramp	n.s. effect on amplitude	n.s. effect on amplitude (HP -65 mV)	Spadoni et al. [205]
100 µM	rat CA1 pyramidal neurons	step pulse 250 ms	-54% amplitude	not assessed	Niespodziany et al. [165]
100 µM	rat CA1 pyramidal neurons	50 mV/s ramp	-58% peak amplitude	-27% amplitude (HP -70 mV)	Yue et al. [251]
100 µM	rat neocortical pyramidal neurons	50 & 100 mV/s ramp	n.s. effect on amplitude	9 mV V_0.5_ left shift of fast steady state inactivation	Colombo et al. [45]
		10 mV/s ramp step pulse 10 s 50 mV/s ramp after 10 s slow steady state inactivation	-34% peak amplitude -26% amplitude 7 mV V_0.5_ left shift		
		50 mV/s ramp after 200 ms depolarizing prepulse	-20% peak amplitude (EC_50_ 28 µM)		
		50 mV/s ramp after 500 ms depolarizing prepulse	-25% peak amplitude (EC_50_ 18 µM)		
100 µM	N1E-115 neuroblastoma cells	slow steady state inactivation 10 s	n.s. effect on V_0.5_	not assessed	Niespodziany et al. [166]
100 µM	tsA201 cells transfected with a Na_v_1.2 mutation	step pulse 200 ms	-88% amplitude (IC_50_ 16 µM)	-40% amplitude (IC_50_ 143 µM, HP -120 mV)	Anderson et al. [8]
50 µM	rat CA1 pyramidal neurons	step pulse 50 ms	n.s. effect on amplitude	-14% amplitude at HP -120 mV	Zeng et al. [253]
		slow steady state inactivation 10 s	8 mV V_0.5_ left shift	-24% amplitude at HP -100 mV	
		entry into slow inactivation 10 s	-40% amplitude	-39% amplitude at HP -80 mV (IC_50_ 73 µM) n.s. V_0.5_ shift of fast steady state inactivation 50 ms 7 mV V_0.5_ left shift of steady state inactivation 500 ms	
4 µM	mouse hippocampal pyramidal neurons with a Na_v_1.6 mutation	step pulse 200 ms	-45% peak amplitude	n.s. effect on amplitude (HP -120 mV)	Baker et al. [18]
100 µM	mouse CA1 pyramidal cells	50 mV/s ramp	-62.5% peak amplitude	not assessed	Kang et al. [114]
	mouse CA1 PV + basket cells	50 mV/s ramp	-78.9% peak amplitude		
100 µM	HEK-293 cells expressing hNa_v_1.6 perfused with ATX-II	step pulse 200 ms	-57.8% amplitude (IC_50_ 60 µM) IC_50_ 48 µM	not assessed	Kahlig et al. [111]
		entry into slow inactivation 8 s			
**PRAX-562**					
100 nM	HEK-293 cells expressing hNa_v_1.6 perfused with ATX-II	step pulse 200 ms	-45% amplitude (IC_50_ 141 nM)		Kahlig et al. [111]
1 µM		entry into slow inactivation 8 s	-72.3% peak amplitude (IC_50_ 317 nM)	-13.8% peak amplitude (IC_50_ 8.4 µM, HP -120 mV) 12 mV V_0.5_ left shift of steady state inactivation 500 ms 3 mV left shift of activation	
**Propofol**					
10 µM	rat neocortical pyramidal neurons	53 mV/s ramp	-75% peak amplitude (IC_50_ 4 µM)	n.s. effect on amplitude (HP -70 mV)	Martella et al. [149]
56 µM	rat medial geniculate body neurons	step pulse 1 s	-45% amplitude	not assessed	Shi et al. [198]
**Ranolazine**					
10 µM 3 µM	NG108-15 cells	70 mV/s ramp 70 mV/s ramp	-58% peak amplitude -41% peak amplitude	15 mV V_0.5_ left shift of fast steady state inactivation 100 ms	Wu et al. [244]
30 µM	tsA201 cells transfected with Na_v_1.1	step pulse 200 ms	-9% peak amplitude (IC_50_ 54 µM)	n.s. effect on V_0.5_ of fast steady state inactivation 100 ms n.s. effect on amplitude (HP -120 mV, IC_50_ 871 µM)	Kahlig et al. [109]
30 µM	nucleated somatic patches of rat CA1 pyramidal neurons	400 mV/s ramp	n.s. effect on amplitude	n.s. effect on amplitude (HP -65 mV)	Park et al. [167]
10 µM	rat cultured hippocampal neurons	slow steady state inactivation 10 s entry into slow inactivation 10 s	10 mV V_0.5_ left shift -31% amplitude	5 mV V_0.5_ left shift of fast steady state inactivation 100 ms	Kahlig et al. [110]
**Riluzole**					
10 µM	rat neocortical pyramidal neurons	step pulse 250 ms	-100% amplitude IC_50_ 2 µM	9 mV V_0.5_ left shift of fast steady state inactivation	Urbani and Belluzzi [228]
		14 mV/s ramp	-100% peak amplitude (identical to 0.3 µM TTX)	(EC_50_ 50 µM; EC_max_ 21 mV)	
10 µM	rat preBötC respiratory pacemaker neurons	20 mV/s ramp	-85% peak amplitude	-8% spike amplitude	Del Negro et al. [53]
1 µM	rat neocortical neurons	60 mV/s ramp	-80% peak amplitude (IC_50_ 550 nM)	-50% amplitude (HP -65 mV)	Spadoni et al. [205]
25 µM	rat isolated suprachiasmatic nucleus neurons	100 mV/s ramp	-100% peak amplitude	not assessed	Kononenko et al. [122]
10 µM	rat CA1 pyramidal neurons	step pulse 250 ms	-56% amplitude	not assessed	Niespodziany et al. [165]
0.5 µM	rat G93A SOD1 mutant cultured motoneurones	10 mV/s ramp	-56% area under the curve	not assessed	Kuo et al. [125]
3 µM	rat preBötC respiratory pacemaker neurons	step pulse 50 ms	-64% amplitude at HP -100 mV -73% amplitude at HP -80 mV -80% amplitude at HP -60 mV	-23% amplitude at HP -100 mV -61% amplitude at HP -80 mV (EC_50_ 2.4 µM) -90% amplitude at HP -60 mV 10 mV V_0.5_ left shift of fast steady state inactivation	Ptak et al. [175]
5 µM	rat mesencephalic layer V neurons	33.3 mV/s ramp	-81% peak amplitude	-11% amplitude (HP -80 mV, EC_50_ 51.6 µM)	Wu et al. [242]
10 µM	rat CA1 pyramidal neurons	50 mV/s ramp	-90% peak amplitude	-30% amplitude (HP -70 mV)	Yue et al. [251]
5 µM 10 µM	rat ventral horn neurones	16 mV/s ramp 16 mV/s ramp	-70% peak amplitude -74% peak amplitude	not assessed	Theiss et al. [221]
10 µM	rat hypoglossal motor neurons	42 mV/s ramp	not quantified in presence of Ca^2+^ blockers	not assessed	Lamanauskas and Nistri [128]
10 µM	mouse superior cervical ganglion neurons	10 mV/s ramp	-70% peak amplitude	-28% amplitude (HP -80 mV)	Lamas et al. [129]
1 µM	cultured embryonic G93A SOD1 mouse cortical neurons	step pulse 80 ms	-48% amplitude	n.s. effect on amplitude (HP -60 mV)	Pieri et al. [173]
10 µM 3 µM	neuroblastoma & glioma NG108-15 cells	70 mV/s ramp 70 mV/s ramp	-55% peak amplitude -34% peak amplitude	13 mV V_0.5_ left shift of fast steady state inactivation	Wu et al. [244]
10 µM	compressed rat DRG neurons	26.7 mV/s ramp	-60% peak amplitude (IC_50_ 4 µM)	n.s. effect on amplitude (HP -80 mV)	Xie et al. [246]
200 µM 500 µM				-59% amplitude (HP -80 mV) -82% amplitude (HP -80 mV)	
10 µM	nucleated somatic patches of rat CA1 pyramidal neurons	400 mV/s ramp 10 mV/s ramp	-73% peak amplitude no I_NaP_ found	-33% amplitude 13 mV V_0.5_ left shift of fast steady state inactivation	Park et al. [167]
20 µM	rat hypoglossal motor neurons	35 mV/s ramp	not quantified	not assessed	Bellingham [25]
10 µM	rat CA3 pyramidal neurons	15 mV/s ramp	-80% peak amplitude	n.s. effect on amplitude (HP -80 mV)	Nakamura et al. [163]
**Rufinamide**					
100 µM	N1E-115 neuroblastoma cells	slow steady state inactivation 10 s	9 mV V_0.5_ right shift	not assessed	Niespodziany et al. [166]
100 µM	Xenopus oocytes with	step pulse 50 ms	n.s. effect on amplitude	n.s. V_0.5_ shift of fast steady state inactivation	Gilchrist et al. [78]
	hNa_v_1.1	entry into slow inactivation 10 s	n.s. effect on amplitude or V_0.5_		
		slow steady state inactivation 10 s	n.s. effect on V_0.5_	8 mV V_0.5_ right shift of activation	
	Xenopus oocytes with hNa_v_1.2 Xenopus oocytes with hNa_v_1.3 Xenopus oocytes with hNa_v_1.6			n.s. V_0.5_ shift of fast steady state inactivation n.s. V_0.5_ shift of fast steady state inactivation 5 mV V_0.5_ right shift of fast steady state inactivation	
**Topiramate**					
100 µM	rat neocortical layer V pyramidal neurons	80 mV/s ramp entry into slow inactivation 800 ms	-40% peak amplitude -65% peak amplitude	7 mV V_0.5_ left shift of fast steady state inactivation 300 ms	Taverna et al. [218]
2 µM	HEK293 cells expressing human Na_v_1.3	step pulse 100 ms	-22% amplitude (EC_50_ 61 nM E_max_ -30%)	1.5 mV V_0.5_ left shift of steady state inactivation 500 ms (EC_50_ 3 µM E_max_ 3 mV)	Sun et al. [215]
**Valproic acid**					
200 µM	dissociated rat neocortical pyramidal neurons	40 mV/s ramp	-80% peak amplitude (EC_50_ 14 µM, EC_max_ -80%)	n.s. effect on amplitude (HP-70 mV)	Taverna et al. [217]
100 µM	rat neocortical pyramidal neurons	53 mV/s ramp	-80% peak amplitude	n.s. effect on amplitude (HP -70 mV)	Martella et al. [149]
10 µM	mouse superior cervical ganglion neurons	10 mV/s ramp	-55% peak amplitude	n.s. effect on amplitude (HP -80 mV)	Lamas et al. [129]
1 mM	HEK-293 cells expressing hNa_v_1.6 perfused with ATX-II	step pulse 200 ms slow entry into slow inactivation 8 s	-2% amplitude -18% peak amplitude	-11% peak amplitude at HP -120 mV	Kahlig et al. [111]
**Zonisamide**					
100 µM	N1E-115 neuroblastoma cells	slow steady state inactivation 10 s	n.s. effect on V_0.5_	not assessed	Niespodziany et al. [166]

### Amiodarone

Amiodarone is a type III antiarrhythmic agent typically used for treatment of ventricular and supraventricular arrhythmia. It predominantly inhibits the human Ether-a-go-go-Related Gene (hERG) potassium channel that mediates a delayed rectified outward potassium current and contributes to the repolarization of cardiac myocytes. The IC_50_ for this effect is 9.8 µM [117]. There is only one study that assessed the effects of amiodarone on I_NaP_ in cortical neurons [205]. Here, the authors saw a > 50% inhibition of I_NaP_ with a ramp protocol at a concentration of 10 µM (Table 1). For Na_V_1.5, the isoform most prevalent in the heart, IC_50_ values were in a similar range [75, 142, 243]. Although amiodarone is a highly lipophilic molecule, its concentration in the brain only reaches 10% of the heart tissue concentration after intravenous administration in the rat [180]. Therefore, it is not suitable for use as an I_NaP_ blocker in neurons in in vivo approaches both in experiments or clinical settings.

### Cannabidiol

Besides the well-known hallucinogenic compound tetrahydrocannabinol, *cannabis sativa* contains more than 100 cannabinods including cannabidiol (CBD). CBD has been used as an anti-seizure drug for patients with Dravet syndrome, a severe epileptic encephalopathy resulting from loss-of-function mutants of Na_v_1.1 [54]. The substance is also known for its anxiolytic and sedative effects [50]. Cannabinoids typically act via the G_i_-protein-coupled cannabinoid receptors CB_1_ in the brain or CB_2_ in periphereral tissues [58]. CBD, at concentrations of 100 nM, acts as a non-competitive antagonist at CB_1_ receptors [222]. It also binds to the serotonin receptor 5-HT1a at the same concentration [188] and activates K_v_7 channels with an EC_50_ of 200 nM [255]. Concerning sodium currents, CBD blocks I_NaP_ in step protocols, but seems to be even more efficient in blocking transient, rather than persistent sodium currents (Table 1). For Na_v_1.7 Huang et al. [98] report preferential block of fast inactivation with slow binding kinetics (see discussion of phenytoin), while for Na_v_1.8 CBD preferentially blocks slow inactivation [254]. Effects of CBD seem to require rather high concentrations: Ghovanloo et al. [76] report CBD to block the transient component (I_NaT_) of all sodium channel isoforms with an IC_50_ of around 3 µM, while Hill et al. [89] estimate the IC_50_ of CBD for brain-expressed sodium channels even tenfold higher. However, this might be an artefact of using plastic instead of glass reservoirs and tubing [255]. Based in these data, CBD should not be used as an CNS I_NaP_ blocker and likely exerts its neurotherapeutic effects via non-sodium channel mediated pathways.

### Carbamazepine

For a long period of time, carbamazepine has been one of the most popular anti-seizure drugs worldwide. This is due to its good efficacy in focal epilepsy and its low costs [172]. It is also a first line drug in other paroxysmal neurological diseases such as episodic ataxia type 1 [131], trigeminal neuralgia [219], paroxysmal extreme pain disorder [63] or secondary dyskinesia [68]. Carbamazepine affects voltage activated calcium channels, especially Ca_v_2.1, at low potency with an IC_50_ of 452 µM [203]. In addition, at 3 µM it suppresses 70% of D-type potassium currents in NG108-15 cells [97].

The main effect of carbamazepine however, is a left shift of the fast inactivation curve of sodium currents at 10–100 µM [87, 195, 215, 226] (Table 1). This effect leads to an earlier inactivation of sodium channels, which then already begins at more negative potentials. Therefore, a reduction of the persistent sodium current should also be expected under the window current hypothesis, which is reflected in step and ramp protocols [111, 215, 226]. However, multiple studies show a lack of action of carbamazepine on slowly inactivating sodium current at concentrations up to 1 mM [87, 166, 195] with the exception of Kahlig et al. [111]. Weighing the evidence, carbamazepine seems to preferentially target I_NaT_, rather than I_NaP_ (Table 1).

### Cenobamate

The latest anti-seizure drug being approved by the European Medicines Agency is cenobamate [61]. It is used as a third-line therapy for multi-drug-resistant focal epilepsy. Cenobamate is known for its potentiation of GABA_A_-mediated currents with EC_50_ values in the range of 42 to 194 µM [194]. At high concentrations it inhibits L-type calcium currents with an IC_50_ of 350 µM and has little effects on K_V_7.1 (IC_50_ 1.3 mM) and K_V_11.1 (IC_50_ 1.8 mM) [7].

Concerning persistent sodium current, cenobamate has been found to block both the non-inactivating and the slowly inactivating current component with an IC_50_ of 50–70 µM [110, 163] (Table 1), which is well below the reported plasma concentration of 170 µM [34]. It also shifts the fast inactivation to the left, but this effect is much less pronounced than its suppressing effect on I_NaP_. The substance could thus be used as a persistent sodium current blocker, but one has to keep in mind that the positive modulation of GABA_A_ receptors occurs at similar concentrations. Thus, systemic effects may be dominated by either of these mechanisms. It is presently difficult to imagine how the substance can isolate specific effects of I_NaP_ in native brain tissue.

### Eslicarbazepine

Eslicarbazepine is an anti-seizure drug that was developed in order to bypass the side effects of carbamazepine and its potentially harmful metabolite carbamazepine-10,11-epoxide [187]. Like carbamazepine, it also affects calcium channels, with a preference for Ca_v_3.2 (a subunit mediating T-type calcium currents) at an IC_50_ of 62 µM [203]. The effects of eslicarbazepine on sodium currents are different from those of carbamazepine. It does not affect the amplitude or fast inactivation of transient sodium currents. Contrary to carbamazepine it shifts the slow inactivation to more hyperpolarized potentials at 300 µM [21, 87, 93] (Table 1). Because of its slow binding kinetics and/or its lack of action on fast inactivation, 300 µM eslicarbazepine does not affect the persistent sodium current when measured with brief voltage steps or fast ramps [21], while afflicting mild effects in slow ramps [55]. Therefore, the substance should be evaluated as a blocker of I_NaP_, in virtue of being an enhancer of slowy inactivating sodium currents. In complex tissues or living organisms, its efficacy on Ca_v_3.2 at similar concentrations should warrant caution.

### Ethosuximide

Ethosuximide is an odd anti-seizure drug used solely to treat absence seizures, while it is not effective against other types of seizures and therefore not commonly employed. Its main mechanism of action is thought to be a partial block of T-type calcium current in thalamic neurons with an EC_50_ of 200 µM [48]. In addition, at 250 µM there is a partial block of the Na^+^/K^+^ ATPase [77]. At concentrations of 20–50 mM I_NaT_ is reduced by ethosuximide in the squid giant axon in a voltage-independent manner [66]. Reliable block of persistent sodium current in neurons can only be obtained at rather high concentrations of 1–10 mM [33, 135] which exceed typical plasma levels of 0.3–0.7 mM [81]. Thus, while it might preferentially effect I_NaP_, ethosuximide cannot be used as an I_NaP_ blocker, at least in complex preparations containing multiple ion channels.

### Gabapentin

Gabapentin is one of the top 10 most prescribed drugs in the world, as it is commonly used for neuropathic pain in polyneuropathies and other chronic pain conditions. Its main mechanism of action is a block of N-type voltage gated calcium channels via an interaction with the α2δ-1 subunit which gabapentin binds with a K_d_ of 59 nM [169]. One study has shown that 5 µM gabapentin blocks I_NaP_ in dorsal root ganglion cells, using a ramp protocol [250] (Table 1). However, DRG neurons express Na_v_1.7, 1.8 and 1.9, i.e. isoforms with peculiar kinetics, which are not dominant in most central nervous neurons. Taken together with the well described effects on calcium channels, gabapentin is probably not a good candidate for being a general blocker of I_NaP_.

### GS967

GS967, now known as Prax330, is a novel compound initially synthesized for treating cardiac arrhythmias [23]. Until now, no clinical trials have been published for this compound, although a phase I trial has been completed (ACTRN12617001512314). Nonetheless, GS967 has been found to be effective in animal models of monogenetic epilepsy and hemiplegic migraine [9, 16, 18]. With regard to possible mechanisms of action, no studies have been published so far examining effects on calcium channels or other molecular targets. However, GS967 reduces I_NaP_ measured with both ramp and short step pulse protocols (Table 1) at concentrations of 0.2–1 µM [9, 240]. It also shifts the fast inactivation curve to more hyperpolarized potentials (Table 1). At this point of time, there is no study evaluating the effect of the drug on slow sodium current inactivation in neurons. Evidence from cardiac slow (2 s) steady state inactivation, however, suggests that GS967 may also be effective in blocking slowly inactivating sodium current. [88]. Taken together, GS967 is a potent blocker of persistent sodium current, and might therefore be a good candidate for further translational studies.

### Lacosamide

Lacosamide has been used to treat focal epilepsy and is also one of the few drugs approved for managing status epilepticus. Recently, however, there has been increased awareness concerning cardiac arrhythmia after administration of lacosamide [247]. Although lacosamide halfs calcium influx via N-type calcium channels (Ca_V_2.2) in cortical neurons at 200 µM [157], its main mechanisms of action appears to involve slowly inactivating sodium currents and/while binding collapsin response mediator protein 2 (CRMP-2) [28], which influences trafficking of Na_v_1.7 [113]. Similar to eslicarbazepine, lacosamide does not affect fast and intermediate inactivation and has only very small effects on I_NaP_ voltage step protocols [111]. However, at 100–250 µM it shifts of the midpoint voltage (V_0.5_) of slow inactivation by 40 mV to the left [62, 87]. This mechanism may underlie the 50% reduction in the ramp protocol observed at 300 µM [227]. Suggested underlying mechanisms are that lacosamide prefers fast inactivation, but has very slow binding kinetics [104] (see discussion of phenytoin), that it preferentially affects channels with slow inactivation [92] or that its interaction with CRMP-2 confers an enhancement of slow inactivation [158]. Keeping in mind the clinically used serum concentration of 20–40 µM [144] lacosamide should be considered as a potent enhancer of slow inactivation. It has, however, lacking effectiveness for I_NaP_ understood as the non-inactivating component in the brief step protocol.

### Lamotrigine

Lamotrigine is currently the best drug for treating focal epilepsy [145, 147], as it is well tolerated and, in some patients, even stabilizes mood. This is why lamotrigine has also entered the realm of psychiatric disease and is currently used for treating bipolar disorder or depression [47]. Another beneficial property of lamotrigine is its efficacy in generalized epilepsy, while other sodium channel blockers tend to aggravate seizures in these patients. This unique profile is linked to a broad range of mechanisms of action: Lamotrigine inhibits high-voltage-activated Ca^2+^ currents with an EC_50_ of 12 µM [209], which in turn reduces the release of glutamate [236]. It also reduces the uptake of serotonin, noradrenaline and dopamine at concentrations of 200–400 µM [204] and, at 100 µM, lamotrigine induces the expression of GABA-A β3 subunits [237]. Lamotrigine seems to bind slowly to sodium channels, which explains the modest effects on the non-inactivating current component at concentrations of 80–100 µM [111, 205] (Table 1). Accordingly, lamotrigine shifts the slow inactivation to more hyperpolarized potentials and exerts inhibition on I_NaT_ amplitude at depolarized membrane potentials [107, 111, 124] (Table 1). These effects occur in a range of 7–40 µM which is comparable to clinical plasma concentrations of 20 µM [118]. In general, the size of these effects is comparatively low (Fig. 3 A and B) and given the effects on calcium- and GABA_A_-channels, it should not be regarded as a specific I_NaP_ blocker.

### Lidocaine

Lidocaine is one of the most popular local anaesthetics and therefore often used to block nerve conduction during surgery. Due to its low bioavailability it is generally not administered orally [52]. In intensive care medicine, intravenous lidocaine has been used for treating ventricular arrhythmia or status epilepticus in rare cases [252]and it is recommended as an anti-arrhythmic drug during resuscitation [202].

Its mechanism of action is mainly based on the block of sodium channels, though it also blocks HCN channels at 20–50 µM [154]. Lidocaine is one of the few drugs whose binding site in VGSC has been well established: its inhibitory effect is primarily caused by disrupting the coupling between the voltage sensors of the sodium channel via long-range stabilization of the third transmembrane domain in the activated state[161].

Most of the studies concerning lidocaine and persistent sodium current have been performed in myocytes [19, 22, 37, 102, 108, 238]. For neuronal channels, lidocaine at high concentration (300 µM—1 mM) does have major effects on both slow and fast sodium current inactivation [195]. However, one study in neurons shows a remarkable effect of lidocaine at 30 nM on the non-inactivating current component in a short pulse protocol [86]. Clinical use cases typically involve lidocaine levels in the range of 1–10 µM [36]. Thus, while the drug appears to be a blocker of I_NaP_, its cardiac side effects restricts its use to preparations in the laboratory.

### NBI-921352

This is a novel compound which was developed as a specific Na_v_1.6 inhibitor by Xenon Pharmaceuticals [106]. Potent effects on I_NaP_ appear quite possible (Table 1), but data on persistent or slowly inactivating components in neurons is yet missing.

### Oxcarbazepine

Like eslicarbazepine, oxcarbazepine is a derivate of carabamazepine. It is used for focal epilepsy, neuropathic pain and sometimes also during alcohol withdrawal [191]. It is one of the few anti-seizure drugs which are not teratogenic [224]. Unlike carabamazepine and eslicarbazepine, it has been shown to also affect voltage-gated calcium channels, particularly N-type, at concentrations of 2–50 µM [208]. In addition, at 10 µM it suppresses 50% of D-type potassium currents [97]. With regard to sodium currents, oxcarbazepine seems to affect both slow and fast inactivation, with a stronger effect on slow inactivation at 10–100 µM (Table 1). It therefore bridges the gap between carbamazepine (predominantly acting on fast inactivation) and eslicarbazepine (predominantly acting on slow inactivation). The observed effects in ramp and step protocols in neuroblastoma/-glioma cells make it quite possible that the drug inhibits I_NaP_ (Table 1). However, there is no data on effects of oxcarbazepine on I_NaP_ in naturally differentiated neurons. This may be a matter of concern, as oxcarbazepine is the only antiepileptic drug which has been shown to reliably destroy glioma cells. In one study on cells derived from patients with brain tumours, the IC_50_ for induction of apoptosis was 45 µM [51]. Out of three studies on oxcarbazepine which are relevant for this review, two were carried out on neuroblastoma/-glioma cells [87, 97], where the drug might induce apoptosis cascades [51]. Hence, more evidence is needed to classify oxcarbazepine with respect to effects on I_NaP_.

### Phenytoin

Phenytoin is one of the oldest anti-seizure and antiarrhythmic drugs [134]. Nowadays, due to its non-linear pharmacokinetics and severe side effects its use is typically restricted to inpatients [74, 179]. Serum levels of phenytoin are typically at 3–10 µM, where it has a 50% inhibitory effect on calcium channels [155]. Being one of the best characterized sodium channel blockers (Table 1), phenytoin does appear to be a prime example of a drug that affects intermediate inactivation [71, 253]. This explains why the drug does not have an effect on I_NaP_ measured with short voltage steps or fast ramps. However, it strikingly inhibits later phases of currents evoked by voltage steps as well as currents evoked by slow ramps (Table 1). It has been suggested that phenytoin exhibits its effects by slowly binding sodium channels in the fast inactivated state [123]. This argument seems to be at odds, however, with the remaining sensitivity of intermediate inactivation to phenytoin after intracellular proteolysis by papain or pronase [253], which completely abolishes fast inactivation [181]. Therefore, if the effect of phenytoin would target fast inactivation after slowly binding to sodium channels, it should not be active after proteolysis. The substance remains active, however, after intracellular application of papain, suggesting that phenytoin does not inhibit sodium currents by affecting fast inactivation [253]. Thus, phenytoin does not directly interact with the fast inactivation gate. Nevertheless, the observed block can be explained by an interaction with the channel which depends on the graded movement of the activating gating charge. In this way, the drug would mimic and compete with natural fast inactivation [123]. Consequently, papain should enhance the speed of phenytoin block, as confirmed by Quandt [176] in neuroblastoma cells, but contested by Zeng et al. [253] in CA1 pyramidal cells. Is I_NaP_ then generally only a byproduct of a failure of intermediate inactivation? Probably not, as other blockers like riluzole or GS967 manage to suppress I_NaP_ measured with early steps or fast ramps. Once again, the term 'persistent’ sodium current is misleading, because it is also applied to slow inactivation patterns at different time scales. Thus, employing phenytoin as an I_NaP_ blocker appears possible, but one has to keep these limitations in mind.

### Propofol

Propofol is a sedative drug commonly used to induce loss of consciousness during narcosis, especially in patients that develop postoperative nausea from volatile anaesthetics. It is typically used at concentrations of 10–50 µM [189]. Propofol is also the last escalation step in order to suppress status epilepticus. At lower concentrations, the drug has addictive properties, like benzodiazepines. It has to be administered intravenously and can induce vasodilation and transient apnoea [189]. Thus, propofol is only used in intensive care settings. The drug predominantly acts on GABA_A_ receptors by binding their β subunits and potentiates GABA induced currents fivefold at 2 µM or even opens the channels directly at 30 µM [84].

Regarding suppression of I_NaP_ propofol is surprisingly potent in neurons at 10–60 µM (Table 1). However, the reported effects on GABA receptors occur in a similar concentration range, again impeding causal analysis in complex preparations. The combination of all known actions of propofol may explain its efficacy in status epilepticus. In no way can it be regarded a specific I_NaP_ blocker. In addition, its low bioavailability and addictiveness limit any prolonged use in outpatients.

### PRAX-562

PRAX-562 is a novel compound specifically synthesised as a persistent sodium current blocker by Praxis Precision Medicines. It was shown to be effective as an antiseizure drug in the maximal electroshock seizure model in mice [111]. Interestingly, amongst all drugs discussed in this review, it is the only substance inducing a small left shift of I_NaT_ activation. Available data from HEK-cells suggest that it acts as an I_NaP_ blocker by modulating both, fast and slow inactivation, but data from neurons are missing, as are data on potential further molecular targets.

### Ranolazine

Ranolazine is a second-line drug in chronic stable angina pectoris and has shown some efficacy in microvascular coronary dysfunction as well as anti-arrhythmic activity [177]. In myocytes, it blocks persistent sodium currents (IC_50_ 6 µM) and delayed rectifier potassium currents (IC_50_ 12 µM) [83]. While ranolazine can cross the blood brain barrier, the CNS concentration only reaches one third of the plasma levels [109]. Ranolazine at 3–30 µM is effective in blocking I_NaP_ measured with ramps and steps [110, 244], while also significantly affecting slow inactivation (Table 1). It does have a higher affinity for I_NaP_ over I_NaT_, therefore making it an attractive persistent sodium current blocker. In systemic applications, however, the aforementioned effects on the heart and the low blood brain barrier passage must be taken into considerations.

### Riluzole

For 28 years, riluzole has been the only approved drug in amyotrophic lateral sclerosis, where it prolongs life expectancy by around 2–3 months [24]. Interestingly, unlike many other drugs on this list, it is not effective in neuropathic pain [73]. This might be due to its low affinity for calcium channels, with IC_50_ values well above 10 µM [24]. However, at clinically used levels of 1–2 µM riluzole does enhance calcium dependent K^+^ currents and reduces presynaptic transmitter release [24].

Traditionally, riluzole has been the most popular blocker of I_NaP_. The drug affects both ramp and step protocols with IC_50_ values well below 10 µM (Table 1). It also has effects on fast inactivation, though these are rather moderate compared to, e.g., carbamazepine (Table 1). Surprisingly, there is no data directly showing effects on slow inactivation, while indirect evidence from slow ramps and other readouts suggests an effect on intermediate inactivation [160]. Riluzole is one of the most useful I_NaP_ blockers, because it does not affect calcium currents at relevant concentrations and its effects on I_NaP_ have been shown in many different types of neurons [53, 163, 228].

### Rufinamide

Rufinamide is a sparsely used anti-seizure drug for patients with Lennox-Gastaut syndrome. It inhibits metabotropic glutamate receptors 5 (mGluR5) at 100 µM, which can be measured by reduced quisqualate-induced phosphoinositol turnover [13]. At the same time, it inhibits slow and fast inactivation of sodium channels, especially Na_v_1.6, at 100 µM. However, ramp or step protocols for the assessment of effects on I_NaP_ are missing in the literature. It has been suggested that rufinamide exerts a preferential effect on intermediate inactivation, similar to phenytoin [138]. In any case, more data is required to assess its effects on I_NaP_ more completely.

### Tetrodotoxin

Tetrodotoxin (TTX) is a poisonous agent found in puffer fish, where it is synthesised by bacteria [164]. It is considered the most effective sodium channel blocker, as it directly occludes sodium ion permeation through the open channel [132]. While transient sodium channels are typically blocked at concentrations of 1 µM, persistent sodium currents are efficiently blocked at lower concentrations of 20–50 nM in rodent brain slices [207, 231, 251]. However, Taddese and Bean [216] found the TTX at very low concentrations of 5 nM has equal effects on transient and persistent currents in tuberomammillary neurons, casting doubt on its specificity for I_NAP_. Therefore, TTX may be a useful tool for experimental work on I_NaP_, but careful controls for effects on I_NaT_ are required in each specific preparation. Notwithstanding, most studies use low micromolar concentrations of TTX to achieve complete absence of sodium currents [70, 163, 212]. Anyway, TTX has no potential for clinical use, due to its well-known capability to paralyse the diaphragm [164].

### Topiramate

Topiramate is typically used in genetic generalized epilepsy, idiopathic intracranial hypertension and in the prevention of migraine episodes. However, its use is often limited by remarkable word-fluency difficulties [159]. At 10 µM, topiramate increases GABA mediated Cl^−^ influx into neurons by 75% [241], it inhibits Ca_v_2.3 with an IC_50_ of 51 µM [127] and exerts weak inhibition of carbonic anhydrases [193]. Concerning I_NaP_, topiramate is a mildly efficient blocker in both step and ramp protocols. In one study [215] topiramate has a very potent effect on non-inactivating sodium current measured with step pulses with an EC_50_ of 61 nM, but the effect size is limited to a maximum of -30%. To our knowledge there is no data showing its effects on slow inactivation. The observed effects are present at clinically relevant concentrations of 2–20 µM [153], but importantly do only exert a partial block of I_NaP_. Thus, topiramate is not a convincing candidate for use as a selective and efficient persistent sodium current blocker.

### Valproic acid

Valproic acid is the first-choice drug for genetic generalized epilepsy [146, 148], but is also used for stabilizing mood in bipolar disorder and preventing episodes of migraine. Due to its high teratogenicity its use in fertile women is strictly controlled in most countries [223]. This severe side-effect might be mediated by epigenomic effects through inhibition of histone deacetylases with IC_50_ values of 0.5–3 mM [82], well within the range of clinically used serum concentrations which range between 0.3–0.7 mM [31]. In addition, valproic acid at 0.5 mM potentiates GABAergic inhibition via a complex modulation of enzymes in GABA metabolism, and also infers with second messenger pathways [105]. Valproate is a potent blocker of persistent sodium current as assessed by ramps and step protocols in neurons (Table 1). These effects occur in the range of 10–100 µM which is well below the clinically used concentrations. Whether it does or does not affect I_NaT_ remains a controversial issue, with more evidence against (see Table 1) than for such an effect [230]. The marked effect on persistent sodium current might explain the efficacy of valproate in treating status epilepticus. However, as valproic acid has particularly many off-target effects, we do not recommend it as a pharmacological tool to isolate I_NaP._

### Zonisamide

Zonisamide is an anti-seizure drug used in focal epilepsy, with particularly widespread application in Asia. It inhibits T-type Ca^2+^ currents and alters the metabolism of dopamine, 5-HT, and acetylcholine [30]. Although it has been described as a sodium channel blocker, this notion is mostly based on data from sea worm axons [190]. In the only study on mammalian channels in mouse derived neuroblastoma cells, zonisamide had no effect on slow inactivation (Table 1). Therefore, it should not be used as an I_NaP_ blocker.

## Summary and recommendations

The very existence of a persistent sodium current as a separate, clearly definable entity is a controversial topic. The lack of clarity may be, at least in part, explained by the misleading word ‘persistent’, which should be understood as: ‘non-inactivating or slowly inactivating voltage-activated sodium current.’ Additional confounding issues are the poorly understood inactivation mechanisms of sodium channels, both from an electrophysiological and structural point of view. The typically employed voltage clamp protocols do not pick up all components of persistent or slowly inactivating sodium current, such that data is often incomplete. Brief voltage steps, in particular, are unable to assess slowly inactivating components. If one wants to characterise the entire effects of a drug on persistent sodium currents, one should also check for alterations of slow inactivation kinetics (notice the lack of respective of data in Fig. 3B). When using ramp protocols, only slow rates of voltage change (≤ 10 mV/s) do span over time periods of tens of seconds and are therefore able to address slow or intermediate inactivation.

Until now, the literature splits clinically employed sodium channel blockers into several types depending on the mechanism of inactivation which they enhance. Carbamazepine is considered as an archetypic fast inactivation enhancer, while lacosamide is considered to be the prototypic slow inactivation enhancer. However, most of the above-described drugs affect all three types of inactivation.

Based on our systematic literature search, we conclude that there is no pharmacological blocker of I_NaP_ that does not somehow affect transient current components. This is not surprising, since I_NaP_ is most likely a result of specific gating properties of the same sodium channels which mediate I_NaT_, rather than a separate molecular subtype. Typically, blockers of I_NaP_ affect I_NaT_ at higher concentrations, such that they can be considered relatively specific as long as low concentrations are applied.

Another point of concern are the off-target effects of the most effective I_NaP_ blockers. Consistently, these drugs target both voltage gated sodium and calcium channels. This is not unexpected, because both channel families have a common evolutionary ancestor [137] and share a major properties of their 3D structure [39]. Taking all these caveats into account, claims that a specific physiological phenomenon is mediated by I_NaP_ should be based on similar effects of more than one drug, or on the additional use of alternative approaches, like genetic manipulation or dynamic voltage clamp [210].

In this review, we show that for CNS neurons GS967 and riluzole are the ‘best’ persistent sodium current blockers in vitro, as they significantly affect non-inactivating sodium current components measured with both ramp protocols and short voltage steps (Fig. 3A). The ‘best’ substance for enhancing intermediate inactivation is phenytoin and for slow inactivation there is lacosamide (Fig. 3B). Substances like NBI-921352 and PRAX-562 show promise for being specific blockers of I_NaP_, but the present evidence is very limited. TTX and lidocaine are very useful tools for pharmacological isolation of I_NaP_, but cannot be used for CNS purposes in vivo. Based on the available evidence, cannabidiol, ethosuximide, gabapentin, rufinamide and zonisamide should not be employed as *bona fide* persistent sodium current blockers either due to missing data or due to lacking potency (Fig. 3). Clinical translation of ranolazine and amiodarone is hindered by low penetrance of the blood–brain-barrier and simultaneous action on cardiac myocytes. High potential for confounding off target effects limits the use of cenobamate, eslicarbazepine, lamotrigine, oxcarbazepine, propofol, topiramate and valproic acid in complex preparations. And last, while still being sodium channel specific, carbamazepine exerts its main effect on fast inactivation.

Wherever possible, future studies should use a combination of brief voltage steps, voltage ramps and steady state inactivation protocols for fast, intermediate and slow inactivation. Step length for brief pulses should be around 50 ms, and they should be repeated at different voltages in order to consider voltage dependent shifts of persistent sodium currents. Ramps should be employed at around 50 mV/s (or even slower when slow inactivation component shall be directly assessed by voltage ramps; see above). TTX subtraction protocols are recommended. Slow steady state inactivation should be assessed by applying steps of 5–10 s duration, intermediate inactivation with 500 ms to 1000 ms steps and fast inactivation with 50 ms to 100 ms steps. Even if we don’t fully understand I_NaP_, we should try to measure it with comparable parameters to facilitate comparisons and to support the further development of therapeutic strategies for conditions involving pathophysiological effects of persistent sodium currents.

## Abbreviations

CBD: cannabidiol
CNS: central nervous system
CRMP-2: collapsin response mediator protein 2
DRG: dorsal root ganglion
EC_50_: half-maximal effect concentration
EC_max_: maximal effect size
EPSP: excitatory postsynaptic potential
hERG: human Ether-a-go-go-Related Gene
HP: holding potential
IC_50_: half-maximal inhibitory concentration
I_NaP_: persistent sodium current
I_NaT_: transient sodium current
IPSP: inhibitory postsynaptic potential
n.s.: not significant
TTX: tetrodotoxin
V_0.5_: midpoint voltage
VGSC: voltage-gated sodium channels

## References

[1] Ahern CA, Payandeh J, Bosmans F, Chanda B (2016) The hitchhiker’s guide to the voltage-gated sodium channel galaxy. J Gen Physiol 147(1):1–24. 10.1085/jgp.201511492 26712848 10.1085/jgp.201511492 PMC4692491

[2] Akopian AN, Souslova V, England S, Okuse K, Ogata N, Ure J et al (1999) The tetrodotoxin-resistant sodium channel SNS has a specialized function in pain pathways. Nat Neurosci 2(6):541–548. 10.1038/9195 10448219 10.1038/9195

[3] Aldrich RW, Corey DP, Stevens CF (1983) A reinterpretation of mammalian sodium channel gating based on single channel recording. Nature 306(5942):436–441. 10.1038/306436a0 6316158 10.1038/306436a0

[4] Alroy G, Su H, Yaari Y (1999) Protein kinase C mediates muscarinic block of intrinsic bursting in rat hippocampal neurons. J Physiology 518(1):71–79. 10.1111/j.1469-7793.1999.0071r.x 10.1111/j.1469-7793.1999.0071r.x PMC2269419 10373690

[5] Alzheimer C, Schwindt P, Crill W (1993) Modal gating of Na+ channels as a mechanism of persistent Na+ current in pyramidal neurons from rat and cat sensorimotor cortex. J Neurosci 13(2):660–673. 10.1523/jneurosci.13-02-00660.1993 8381170 10.1523/JNEUROSCI.13-02-00660.1993 PMC6576639

[6] Aman TK, Grieco-Calub TM, Chen C, Rusconi R, Slat EA, Isom LL et al (2009) Regulation of persistent Na current by interactions between beta subunits of voltage-gated Na channels. J Neurosci 29(7):2027–2042. 10.1523/jneurosci.4531-08.2009 19228957 10.1523/JNEUROSCI.4531-08.2009 PMC2667244

[7] Amuzescu BC, Dan Corlan A, Radu BM (2023) Inhibitory effects of cenobamate on multiple human cardiac ion channels and possible arrhythmogenic consequences. PREPRINT (Version 1) available at Research Square. 10.21203/rs.3.rs-3735338/v1 10.3390/biom14121582 PMC11674187 39766288

[8] Anderson LL, Thompson CH, Hawkins NA, Nath RD, Petersohn AA, Rajamani S et al (2014) Antiepileptic activity of preferential inhibitors of persistent sodium current. Epilepsia 55(8):1274–1283. 10.1111/epi.12657 24862204 10.1111/epi.12657 PMC4126848

[9] Anderson LL, Hawkins NA, Thompson CH, Kearney JA, George AL Jr (2017) Unexpected Efficacy of a Novel Sodium Channel Modulator in Dravet Syndrome. Sci Rep 7(1):1682. 10.1038/s41598-017-01851-9 28490751 10.1038/s41598-017-01851-9 PMC5431801

[10] Armstrong CM (2006) Na channel inactivation from open and closed states. Proc Natl Acad Sci 103(47):17991–17996. 10.1073/pnas.0607603103 17101981 10.1073/pnas.0607603103 PMC1693860

[11] Armstrong CM, Bezanilla F (1977) Inactivation of the sodium channel. II. Gating current experiments. J Gen Physiol 70(5):567–590. 10.1085/jgp.70.5.567 591912 10.1085/jgp.70.5.567 PMC2228472

[12] Armstrong CM, Gilly WF. [5] Access resistance and space clamp problems associated with whole-cell patch clamping. Methods Enzymol. 207: Academic Press; 1992. p. 100–122 10.1016/0076-6879(92)07007-B 10.1016/0076-6879(92)07007-b 1528114

[13] Arroyo S (2007) Rufinamide. Neurotherapeutics 4(1):155–162. 10.1016/j.nurt.2006.11.006 17199032 10.1016/j.nurt.2006.11.006 PMC7479703

[14] Astman N, Gutnick MJ, Fleidervish IA (1998) Activation of protein kinase C increases neuronal excitability by regulating persistent Na+ current in mouse neocortical slices. J Neurophysiol 80(3):1547–1551. 10.1152/jn.1998.80.3.1547 9744958 10.1152/jn.1998.80.3.1547

[15] Astman N, Gutnick MJ, Fleidervish IA (2006) Persistent sodium current in layer 5 neocortical neurons is primarily generated in the proximal axon. J Neurosci 26(13):3465–3473. 10.1523/jneurosci.4907-05.2006 16571753 10.1523/JNEUROSCI.4907-05.2006 PMC6673860

[16] Auffenberg E, Hedrich UB, Barbieri R, Miely D, Groschup B, Wuttke TV et al (2021) Hyperexcitable interneurons trigger cortical spreading depression in an Scn1a migraine model. J Clin Invest 131(21):e142202. 10.1172/jci142202 34546973 10.1172/JCI142202 PMC8553559

[17] Azouz R, Jensen MS, Yaari Y (1996) Ionic basis of spike after-depolarization and burst generation in adult rat hippocampal CA1 pyramidal cells. J Physiol 492(1):211–223. 10.1113/jphysiol.1996.sp021302 8730596 10.1113/jphysiol.1996.sp021302 PMC1158874

[18] Baker EM, Thompson CH, Hawkins NA, Wagnon JL, Wengert ER, Patel MK et al (2018) The novel sodium channel modulator GS-458967 (GS967) is an effective treatment in a mouse model of SCN8A encephalopathy. Epilepsia 59(6):1166–1176. 10.1111/epi.14196 29782051 10.1111/epi.14196 PMC6142814

[19] Balser JR, Nuss HB, Romashko DN, Marban E, Tomaselli GF (1996) Functional consequences of lidocaine binding to slow-inactivated sodium channels. J Gen Physiol 107(5):643–658. 10.1085/jgp.107.5.643 8740377 10.1085/jgp.107.5.643 PMC2217016

[20] Barbieri R, Bertelli S, Pusch M, Gavazzo P (2019) Late sodium current blocker GS967 inhibits persistent currents induced by familial hemiplegic migraine type 3 mutations of the SCN1A gene. J Headache Pain 20(1):107. 10.1186/s10194-019-1056-2 31730442 10.1186/s10194-019-1056-2 PMC6858687

[21] Bayraktar E, Liu Y, Sonnenberg L, Hedrich UBS, Sara Y, Eltokhi A et al (2022) In vitro effects of eslicarbazepine (S-licarbazepine) as a potential precision therapy on SCN8A variants causing neuropsychiatric disorders. Br J Pharmacol 180(8):1038–1055. 10.1111/bph.15981 36321697 10.1111/bph.15981

[22] Bean BP, Cohen CJ, Tsien RW (1983) Lidocaine block of cardiac sodium channels. J Gen Physiol 81(5):613–642 6306139 10.1085/jgp.81.5.613 PMC2216565

[23] Belardinelli L, Liu G, Smith-Maxwell C, Wang W-Q, El-Bizri N, Hirakawa R et al (2013) A Novel, Potent, and Selective Inhibitor of Cardiac Late Sodium Current Suppresses Experimental Arrhythmias. J Pharmacol Exp Ther 344(1):23–32. 10.1124/jpet.112.198887 23010360 10.1124/jpet.112.198887

[24] Bellingham MC (2011) A review of the neural mechanisms of action and clinical efficiency of riluzole in treating amyotrophic lateral sclerosis: what have we learned in the last decade? CNS Neurosci Ther 17(1):4–31. 10.1111/j.1755-5949.2009.00116.x 20236142 10.1111/j.1755-5949.2009.00116.x PMC6493865

[25] Bellingham MC (2013) Pre- and postsynaptic mechanisms underlying inhibition of hypoglossal motor neuron excitability by riluzole. J Neurophysiol 110(5):1047–1061. 10.1152/jn.00587.2012 23741042 10.1152/jn.00587.2012

[26] Berger T, Lüscher HR (2004) Associative somatodendritic interaction in layer V pyramidal neurons is not affected by the antiepileptic drug lamotrigine. Eur J Neurosci 20(6):1688–1693. 10.1111/j.1460-9568.2004.03617.x 15355337 10.1111/j.1460-9568.2004.03617.x

[27] Bevan MD, Wilson CJ (1999) Mechanisms underlying spontaneous oscillation and rhythmic firing in rat subthalamic neurons. J Neurosci 19(17):7617–7628. 10.1523/JNEUROSCI.19-17-07617.1999 10460267 10.1523/JNEUROSCI.19-17-07617.1999 PMC6782508

[28] Beyreuther BK, Freitag J, Heers C, Krebsfänger N, Scharfenecker U, Stöhr T (2007) Lacosamide: a review of preclinical properties. CNS Drug Rev 13(1):21–42. 10.1111/j.1527-3458.2007.00001.x 17461888 10.1111/j.1527-3458.2007.00001.x PMC6494128

[29] Bezanilla F, Armstrong CM (1977) Inactivation of the sodium channel. I. Sodium current experiments. J Gen Physiol 70(5):549–566. 10.1085/jgp.70.5.549 591911 10.1085/jgp.70.5.549 PMC2228478

[30] Biton V (2007) Clinical Pharmacology and Mechanism of Action of Zonisamide. Clin Neuropharmacol 30:4. 10.1097/wnf.0b013e3180413d7d 10.1097/wnf.0b013e3180413d7d 17762320

[31] Bowden CL, Janicak PG, Orsulak P, Swann AC, Davis JM, Calabrese JR et al (1996) Relation of serum valproate concentration to response in mania. Am J Psychiatry 153(6):765–770. 10.1176/ajp.153.6.765 8633687 10.1176/ajp.153.6.765

[32] Brocard C, Plantier V, Boulenguez P, Liabeuf S, Bouhadfane M, Viallat-Lieutaud A et al (2016) Cleavage of Na(+) channels by calpain increases persistent Na(+) current and promotes spasticity after spinal cord injury. Nat Med 22(4):404–411. 10.1038/nm.4061 26974309 10.1038/nm.4061

[33] Broicher T, Seidenbecher T, Meuth P, Munsch T, Meuth SG, Kanyshkova T et al (2007) T-current related effects of antiepileptic drugs and a Ca2+ channel antagonist on thalamic relay and local circuit interneurons in a rat model of absence epilepsy. Neuropharmacology 53(3):431–446. 10.1016/j.neuropharm.2007.05.030 17675191 10.1016/j.neuropharm.2007.05.030

[34] Brown PC. 212839Orig1s000. NON-CLINICAL REVIEW(S), U.S. Food and Drug Administration

[35] Bunton-Stasyshyn RKA, Wagnon JL, Wengert ER, Barker BS, Faulkner A, Wagley PK et al (2019) Prominent role of forebrain excitatory neurons in SCN8A encephalopathy. Brain 142(2):362–375. 10.1093/brain/awy324 30601941 10.1093/brain/awy324 PMC6351781

[36] Butterwick KJ, Goldman MP, Sriprachya-Anunt S (1999) Lidocaine levels during the first two hours of infiltration of dilute anesthetic solution for tumescent liposuction: rapid versus slow delivery. Dermatol Surg 25(9):681–685. 10.1046/j.1524-4725.1999.98275.x 10491056 10.1046/j.1524-4725.1999.98275.x

[37] Carmeliet E, Saikawa T (1982) Shortening of the action potential and reduction of pacemaker activity by lidocaine, quinidine, and procainamide in sheep cardiac purkinje fibers. An effect on Na or K currents? Circ Res 50(2):257–272. 10.1161/01.res.50.2.257 6276042 10.1161/01.res.50.2.257

[38] Carter BC, Giessel AJ, Sabatini BL, Bean BP (2012) Transient sodium current at subthreshold voltages: activation by EPSP waveforms. Neuron 75(6):1081–1093. 10.1016/j.neuron.2012.08.033 22998875 10.1016/j.neuron.2012.08.033 PMC3460524

[39] Catterall WA, TM, Swanson (2015) Structural Basis for Pharmacology of Voltage-Gated Sodium and Calcium Channels. Mol Pharmacol 88(1):141. 10.1124/mol.114.097659 10.1124/mol.114.097659 PMC4468632 25848093

[40] Cestèle S, Scalmani P, Rusconi R, Terragni B, Franceschetti S, Mantegazza M (2008) Self-Limited Hyperexcitability: Functional Effect of a Familial Hemiplegic Migraine Mutation of the Nav1.1 (SCN1A) Na+ Channel. J Neurosci 28(29):7273–7283. 10.1523/jneurosci.4453-07.2008 10.1523/JNEUROSCI.4453-07.2008 PMC2721955 18632931

[41] Chandler WK, Meves H (1970) Evidence for two types of sodium conductance in axons perfused with sodium fluoride solution. J Physiol 211(3):653–678. 10.1113/jphysiol.1970.sp009298 5501056 10.1113/jphysiol.1970.sp009298 PMC1396075

[42] Chao TI, Alzheimer C (1995) Effects of phenytoin on the persistent Na+ current of mammalian CNS neurones. NeuroReport 6(13):1778–1780. 10.1097/00001756-199509000-00017 8541480 10.1097/00001756-199509000-00017

[43] Chen Y, Yu FH, Sharp EM, Beacham D, Scheuer T, Catterall WA (2008) Functional properties and differential neuromodulation of Na(v)1.6 channels. Mol Cell Neurosci 38(4):607–615. 10.1016/j.mcn.2008.05.009 18599309 10.1016/j.mcn.2008.05.009 PMC3433175

[44] Clatot J, Hoshi M, Wan X, Liu H, Jain A, Shinlapawittayatorn K et al (2017) Voltage-gated sodium channels assemble and gate as dimers. Nat Commun 8(1):2077. 10.1038/s41467-017-02262-0 29233994 10.1038/s41467-017-02262-0 PMC5727259

[45] Colombo E, Franceschetti S, Avanzini G, Mantegazza M (2013) Phenytoin inhibits the persistent sodium current in neocortical neurons by modifying its inactivation properties. PLoS One 8(1):e55329. 10.1371/journal.pone.0055329 23383157 10.1371/journal.pone.0055329 PMC3558486

[46] Correa AM, Bezanilla F (1994) Gating of the squid sodium channel at positive potentials: II. Single channels reveal two open states. Biophys J 66(6):1864–1878. 10.1016/S0006-3495(94)80980-4 8075324 10.1016/S0006-3495(94)80980-4 PMC1275912

[47] Costa B, Vale N (2023) Understanding Lamotrigine&rsquo;s Role in the CNS and Possible Future Evolution. Int J Mol Sci 24(7):6050. 10.3390/ijms24076050 37047022 10.3390/ijms24076050 PMC10093959

[48] Coulter DA, Huguenard JR, Prince DA (1989) Characterization of ethosuximide reduction of low-threshold calcium current in thalamic neurons. Ann Neurol 25(6):582–593. 10.1002/ana.410250610 2545161 10.1002/ana.410250610

[49] Crill WE (1996) Persistent Sodium Current in Mammalian Central Neurons. Annu Rev Physiol 58(1):349–362. 10.1146/annurev.ph.58.030196.002025 8815799 10.1146/annurev.ph.58.030196.002025

[50] Crippa JA, Guimarães FS, Campos AC, Zuardi AW (2018) Translational Investigation of the Therapeutic Potential of Cannabidiol (CBD): Toward a New Age. Front Immunol 9:2009. 10.3389/fimmu.2018.02009 30298064 10.3389/fimmu.2018.02009 PMC6161644

[51] Dao Trong P, Jungwirth G, Unterberg A, Herold-Mende C, Warta R (2023) The Antiepileptic Drug Oxcarbazepine Inhibits the Growth of Patient-Derived Isocitrate Dehydrogenase Mutant Glioma Stem-like Cells. Cells 12(8):1200. 10.3390/cells12081200 37190109 10.3390/cells12081200 PMC10136933

[52] de Boer AG, Breimer DD, Mattie H, Pronk J, Gubbens-Stibbe JM (1979) Rectal bioavailability of lidocaine in man: Partial avoidance of “first-pass” metabolism. Clin Pharmacol Ther 26(6):701–709. 10.1002/cpt1979266701 498711 10.1002/cpt1979266701

[53] Del Negro CA, Morgado-Valle C, Feldman JL (2002) Respiratory Rhythm: An Emergent Network Property? Neuron 34(5):821–830. 10.1016/S0896-6273(02)00712-2 12062027 10.1016/s0896-6273(02)00712-2

[54] Devinsky O, Cross JH, Laux L, Marsh E, Miller I, Nabbout R et al (2017) Trial of Cannabidiol for Drug-Resistant Seizures in the Dravet Syndrome. N Engl J Med 376(21):2011–2020. 10.1056/NEJMoa1611618 28538134 10.1056/NEJMoa1611618

[55] Doeser A, Soares-da-Silva P, Beck H, Uebachs M (2014) The effects of eslicarbazepine on persistent Na+ current and the role of the Na+ channel β subunits. Epilepsy Res 108(2):202–211. 10.1016/j.eplepsyres.2013.11.022 10.1016/j.eplepsyres.2013.11.022 24368131

[56] Dong H, Fan YH, Wang YY, Wang WT, Hu SJ (2008) Lidocaine suppresses subthreshold oscillations by inhibiting persistent Na(+) current in injured dorsal root ganglion neurons. Physiol Res 57(4):639–645. 10.33549/physiolres.931164 17705679 10.33549/physiolres.931164

[57] Dubois JM, Bergman C (1975) Late sodium current in the node of Ranvier. Pflugers Arch 357(1–2):145–148. 10.1007/bf00584552 1080274 10.1007/BF00584552

[58] Elphick MR, Egertova M (2001) The neurobiology and evolution of cannabinoid signalling. Philos Trans R Soc Lond B Biol Sci 356(1407):381–408. 10.1098/rstb.2000.0787 11316486 10.1098/rstb.2000.0787 PMC1088434

[59] Errington AC, Stöhr T, Heers C, Lees G (2008) The Investigational Anticonvulsant Lacosamide Selectively Enhances Slow Inactivation of Voltage-Gated Sodium Channels. Mol Pharmacol 73(1):157–169. 10.1124/mol.107.039867 17940193 10.1124/mol.107.039867

[60] Estacion M, Gasser A, Dib-Hajj SD, Waxman SG (2010) A sodium channel mutation linked to epilepsy increases ramp and persistent current of Nav1.3 and induces hyperexcitability in hippocampal neurons. Exp Neurol 224(2):362–368. 10.1016/j.expneurol.2010.04.012 20420834 10.1016/j.expneurol.2010.04.012

[61] European-Medicines-Agency. Ontozry 2021. Available from: https://www.ema.europa.eu/en/medicines/human/EPAR/ontozry

[62] Feng YC, Howrigan DP, Abbott LE, Tashman K, Cerrato F, Singh T, Heyne H, Byrnes A, Churchhouse C, Watts N, Solomonson M (2019) Ultra-Rare Genetic Variation in the Epilepsies: A Whole-Exome Sequencing Study of 17, 606 Individuals. Am J Hum Genet 105(2):267–282. 10.1016/j.ajhg.2019.05.020 31327507 10.1016/j.ajhg.2019.05.020 PMC6698801

[63] Fertleman CR, Baker MD, Parker KA, Moffatt S, Elmslie FV, Abrahamsen B et al (2006) SCN9A mutations in paroxysmal extreme pain disorder: allelic variants underlie distinct channel defects and phenotypes. Neuron 52(5):767–774. 10.1016/j.neuron.2006.10.006 17145499 10.1016/j.neuron.2006.10.006

[64] Fleidervish IA, Gutnick MJ (1996) Kinetics of slow inactivation of persistent sodium current in layer V neurons of mouse neocortical slices. J Neurophysiol 76(3):2125–2130. 10.1152/jn.1996.76.3.2125 8890326 10.1152/jn.1996.76.3.2125

[65] Fleidervish IA, Libman L, Katz E, Gutnick MJ (2008) Endogenous polyamines regulate cortical neuronal excitability by blocking voltage-gated Na+ channels. Proc Natl Acad Sci 105(48):18994–18999. 10.1073/pnas.0803464105 10.1073/pnas.0803464105 PMC2596226 19020082

[66] Fohlmeister JF, Adelman WJ Jr, Brennan JJ (1984) Excitable channel currents and gating times in the presence of anticonvulsants ethosuximide and valproate. J Pharmacol Exp Ther 230(1):75–81 6086880

[67] Franceschetti S, Taverna S, Sancini G, Panzica F, Lombardi R, Avanzini G (2000) Protein kinase C-dependent modulation of Na+ currents increases the excitability of rat neocortical pyramidal neurones. J Physiology 528(Pt 2):291–304. 10.1111/j.1469-7793.2000.00291.x 10.1111/j.1469-7793.2000.00291.x PMC2270127 11034619

[68] Freiha J, Riachi N, Chalah MA, Zoghaib R, Ayache SS, Ahdab R (2020) Paroxysmal Symptoms in Multiple Sclerosis-A Review of the Literature. J Clin Med 9(10):3100. 10.3390/jcm9103100 32992918 10.3390/jcm9103100 PMC7600828

[69] French CR, Gage PW (1985) A threshold sodium current in pyramidal cells in rat hippocampus. Neurosci Lett 56(3):289–193. 10.1016/0304-3940(85)90257-5 2410817 10.1016/0304-3940(85)90257-5

[70] French CR, Sah P, Buckett KJ, Gage PW (1990) A voltage-dependent persistent sodium current in mammalian hippocampal neurons. J Gen Physiol 95(6):1139–1157. 10.1085/jgp.95.6.1139 10.1085/jgp.95.6.1139 PMC2216358 2374000

[71] French CR, Zeng Z, Williams DA, Hill-Yardin EL, O’Brien TJ (2015) Properties of an intermediate-duration inactivation process of the voltage-gated sodium conductance in rat hippocampal CA1 neurons. J Neurophysiol 115(2):790–802. 10.1152/jn.01000.2014 26538613 10.1152/jn.01000.2014

[72] Fricker D, Miles R (2000) EPSP amplification and the precision of spike timing in hippocampal neurons. Neuron 28(2):559–569. 10.1016/s0896-6273(00)00133-1 11144364 10.1016/s0896-6273(00)00133-1

[73] Galer BS, Twilling LL, Harle J, Cluff RS, Friedman E, Rowbotham MC (2000) Lack of efficacy of riluzole in the treatment of peripheral neuropathic pain conditions. Neurology 55(7):971–975. 10.1212/wnl.55.7.971 11061253 10.1212/wnl.55.7.971

[74] Gallop K (2010) Review article: phenytoin use and efficacy in the ED. Emerg Med Australas 22(2):108–118. 10.1111/j.1742-6723.2010.01269.x 20534046 10.1111/j.1742-6723.2010.01269.x

[75] Ghovanloo MR, Abdelsayed M, Ruben PC (2016) Effects of amiodarone and n-desethylamiodarone on cardiac voltage-gated sodium channels. Front Pharmacol 7. 10.3389/fphar.2016.00039 10.3389/fphar.2016.00039 PMC4771766 26973526

[76] Ghovanloo M-R, Shuart NG, Mezeyova J, Dean RA, Ruben PC, Goodchild SJ (2018) Inhibitory effects of cannabidiol on voltage-dependent sodium currents. J Biol Chem 293(43):16546–16558. 10.1074/jbc.RA118.004929 30219789 10.1074/jbc.RA118.004929 PMC6204917

[77] Gilbert JC, Scott AK, Wyllie MG (1974) Proceedings: Effects of ethosuximide on adenosine triphosphatase activities of some subcellular fractions prepared from rat cerebral cortex. Br J Pharmacol 50(3):452–453 PMC1776713 4277617

[78] Gilchrist J, Dutton S, Diaz-Bustamante M, McPherson A, Olivares N, Kalia J et al (2014) Nav1.1 modulation by a novel triazole compound attenuates epileptic seizures in rodents. ACS Chem Biol 9(5):1204–1212. 10.1021/cb500108p 24635129 10.1021/cb500108p PMC4027953

[79] Gorelova N, Seamans JK (2015) Cell-attached single-channel recordings in intact prefrontal cortex pyramidal neurons reveal compartmentalized D1/D5 receptor modulation of the persistent sodium current. Front Neural Circuits 9. 10.3389/fncir.2015.00004 10.3389/fncir.2015.00004 PMC4325928 25729354

[80] Gorelova NA, Yang CR (2000) Dopamine D1/D5 receptor activation modulates a persistent sodium current in rat prefrontal cortical neurons in vitro. J Neurophysiol 84(1):75–87. 10.1152/jn.2000.84.1.75 10899185 10.1152/jn.2000.84.1.75

[81] Gören MZ, Onat F (2007) Ethosuximide: From Bench to Bedside. CNS Drug Rev 13(2):224–239. 10.1111/j.1527-3458.2007.00009.x 17627674 10.1111/j.1527-3458.2007.00009.x PMC6528725

[82] Göttlicher M, Minucci S, Zhu P, Krämer OH, Schimpf A, Giavara S et al (2001) Valproic acid defines a novel class of HDAC inhibitors inducing differentiation of transformed cells. EMBO J 20(24):6969–6978. 10.1093/emboj/20.24.6969 11742974 10.1093/emboj/20.24.6969 PMC125788

[83] Gupta T, Khera S, Kolte D, Aronow WS, Iwai S (2015) Antiarrhythmic properties of ranolazine: A review of the current evidence. Int J Cardiol 187:66–74. 10.1016/j.ijcard.2015.03.324 25828315 10.1016/j.ijcard.2015.03.324

[84] Hales TG, Lambert JJ (1991) The actions of propofol on inhibitory amino acid receptors of bovine adrenomedullary chromaffin cells and rodent central neurones. Br J Pharmacol 104(3):619–628. 10.1111/j.1476-5381.1991.tb12479.x 1665745 10.1111/j.1476-5381.1991.tb12479.x PMC1908220

[85] Hamada MS, Kole MH (2015) Myelin loss and axonal ion channel adaptations associated with gray matter neuronal hyperexcitability. J Neurosci 35(18):7272–7286. 10.1523/jneurosci.4747-14.2015 25948275 10.1523/JNEUROSCI.4747-14.2015 PMC4420788

[86] Hammarstrom AK, Gage PW (1998) Inhibition of oxidative metabolism increases persistent sodium current in rat CA1 hippocampal neurons. J Physiol 510(3):735–741. 10.1111/j.1469-7793.1998.735bj.x 9660889 10.1111/j.1469-7793.1998.735bj.x PMC2231084

[87] Hebeisen S, Pires N, Loureiro AI, Bonifácio MJ, Palma N, Whyment A et al (2015) Eslicarbazepine and the enhancement of slow inactivation of voltage-gated sodium channels: a comparison with carbamazepine, oxcarbazepine and lacosamide. Neuropharmacology 89:122–135. 10.1016/j.neuropharm.2014.09.008 25242737 10.1016/j.neuropharm.2014.09.008

[88] Hézső T, Naveed M, Dienes C, Kiss D, Prorok J, Árpádffy-Lovas T et al (2021) Mexiletine-like cellular electrophysiological effects of GS967 in canine ventricular myocardium. Sci Rep 11(1):9565. 10.1038/s41598-021-88903-3 33953276 10.1038/s41598-021-88903-3 PMC8100105

[89] Hill AJ, Jones NA, Smith I, Hill CL, Williams CM, Stephens GJ et al (2014) Voltage-gated sodium (NaV) channel blockade by plant cannabinoids does not confer anticonvulsant effects per se. Neurosci Lett 566:269–274. 10.1016/j.neulet.2014.03.013 24642454 10.1016/j.neulet.2014.03.013

[90] Hodge RD, Bakken TE, Miller JA, Smith KA, Barkan ER, Graybuck LT et al (2019) Conserved cell types with divergent features in human versus mouse cortex. Nature 573(7772):61–68. 10.1038/s41586-019-1506-7 31435019 10.1038/s41586-019-1506-7 PMC6919571

[91] Hodgkin AL, Huxley AF (1952) A quantitative description of membrane current and its application to conduction and excitation in nerve. J Physiol 117(4):500–544. 10.1113/jphysiol.1952.sp004764 12991237 10.1113/jphysiol.1952.sp004764 PMC1392413

[92] Holtkamp D, Opitz T, Niespodziany I, Wolff C, Beck H (2017) Activity of the anticonvulsant lacosamide in experimental and human epilepsy via selective effects on slow Na(+) channel inactivation. Epilepsia 58(1):27–41. 10.1111/epi.13602 27864845 10.1111/epi.13602

[93] Holtkamp D, Opitz T, Hebeisen S, Soares-da-Silva P, Beck H (2018) Effects of eslicarbazepine on slow inactivation processes of sodium channels in dentate gyrus granule cells. Epilepsia 59(8):1492–1506. 10.1111/epi.14504 29953587 10.1111/epi.14504

[94] Horn EM, Waldrop TG (2000) Hypoxic augmentation of fast-inactivating and persistent sodium currents in rat caudal hypothalamic neurons. J Neurophysiol 84(5):2572–2581. 10.1152/jn.2000.84.5.2572 11067999 10.1152/jn.2000.84.5.2572

[95] Hsu CL, Zhao X, Milstein AD, Spruston N (2018) Persistent Sodium Current Mediates the Steep Voltage Dependence of Spatial Coding in Hippocampal Pyramidal Neurons. Neuron 99(1):147-162.e8. 10.1016/j.neuron.2018.05.025 29909995 10.1016/j.neuron.2018.05.025 PMC6179354

[96] Hu W, Tian C, Li T, Yang M, Hou H, Shu Y (2009) Distinct contributions of Nav1.6 and Nav1.2 in action potential initiation and backpropagation. Nat Neurosci 12(8):996–1002. 10.1038/nn.2359 19633666 10.1038/nn.2359

[97] Huang C-W, Huang C-C, Lin M-W, Tsai J-J, Wu S-N (2008) The synergistic inhibitory actions of oxcarbazepine on voltage-gated sodium and potassium currents in differentiated NG108–15 neuronal cells and model neurons. Int J Neuropsychopharmacol 11(5):597–610. 10.1017/S1461145707008346 18184444 10.1017/S1461145707008346

[98] Huang J, Fan X, Jin X, Jo S, Zhang HB, Fujita A et al (2023) Cannabidiol inhibits Nav channels through two distinct binding sites. Nat Commun 14(1):3613. 10.1038/s41467-023-39307-6 37330538 10.1038/s41467-023-39307-6 PMC10276812

[99] Hutcheon B, Yarom Y (2000) Resonance, oscillation and the intrinsic frequency preferences of neurons. Trends Neurosci 23(5):216–222. 10.1016/s0166-2236(00)01547-2 10782127 10.1016/s0166-2236(00)01547-2

[100] Igelström KM, Heyward PM (2012) The antidepressant drug fluoxetine inhibits persistent sodium currents and seizure-like events. Epilepsy Res 101(1):174–181. 10.1016/j.eplepsyres.2012.03.019 22520760 10.1016/j.eplepsyres.2012.03.019

[101] Ilin V, Malyshev A, Wolf F, Volgushev M (2013) Fast Computations in Cortical Ensembles Require Rapid Initiation of Action Potentials. J Neurosci 33(6):2281–2292. 10.1523/jneurosci.0771-12.2013 23392659 10.1523/JNEUROSCI.0771-12.2013 PMC3964617

[102] Inomata N, Ishihara T, Akaike N (1989) Different time courses of the blockade of sodium current by lignocaine and SUN 1165 in single myocytes isolated from guinea-pig atrium. Br J Pharmacol 98(1):149–154. 10.1111/j.1476-5381.1989.tb16875.x 2553185 10.1111/j.1476-5381.1989.tb16875.x PMC1854666

[103] Iwai Y, Shibuya K, Misawa S, Sekiguchi Y, Watanabe K, Amino H et al (2016) Axonal Dysfunction Precedes Motor Neuronal Death in Amyotrophic Lateral Sclerosis. PLoS One 11(7):e0158596. 10.1371/journal.pone.0158596 27383069 10.1371/journal.pone.0158596 PMC4934877

[104] Jo S, Bean BP (2017) Lacosamide Inhibition of Nav1.7 Voltage-Gated Sodium Channels: Slow Binding to Fast-Inactivated States. Mol Pharmacol 91(4):277–286. 10.1124/mol.116.106401 28119481 10.1124/mol.116.106401 PMC5363714

[105] Johannessen CU (2000) Mechanisms of action of valproate: a commentatory. Neurochem Int 37(2–3):103–110. 10.1016/s0197-0186(00)00013-9 10812195 10.1016/s0197-0186(00)00013-9

[106] Johnson JP, Focken T, Khakh K, Tari PK, Dube C, Goodchild SJ, et al (2022) NBI-921352, a first-in-class, Na(V)1.6 selective, sodium channel inhibitor that prevents seizures in Scn8a gain-of-function mice, and wild-type mice and rats. Elife 11 10.7554/eLife.72468 10.7554/eLife.72468 PMC8903829 35234610

[107] Jones PJ, Merrick EC, Batts TW, Hargus NJ, Wang Y, Stables JP et al (2009) Modulation of sodium channel inactivation gating by a novel lactam: implications for seizure suppression in chronic limbic epilepsy. J Pharmacol Exp Ther 328(1):201–212. 10.1124/jpet.108.144709 18952887 10.1124/jpet.108.144709 PMC2685906

[108] Ju YK, Saint DA, Gage PW (1996) Hypoxia increases persistent sodium current in rat ventricular myocytes. J Physiol 497(2):337–347. 10.1113/jphysiol.1996.sp021772 8961179 10.1113/jphysiol.1996.sp021772 PMC1160988

[109] Kahlig KM, Lepist I, Leung K, Rajamani S, George AL (2010) Ranolazine selectively blocks persistent current evoked by epilepsy-associated Naν1.1 mutations. Br J Pharmacol 161(6):1414–1426. 10.1111/j.1476-5381.2010.00976.x 20735403 10.1111/j.1476-5381.2010.00976.x PMC3000664

[110] Kahlig KM, Hirakawa R, Liu L, George AL, Belardinelli L, Rajamani S (2014) Ranolazine Reduces Neuronal Excitability by Interacting with Inactivated States of Brain Sodium Channels. Mol Pharmacol 85(1):162–174. 10.1124/mol.113.088492 24202911 10.1124/mol.113.088492

[111] Kahlig KM, Scott L, Hatch RJ, Griffin A, Martinez Botella G, Hughes ZA et al (2022) The novel persistent sodium current inhibitor PRAX-562 has potent anticonvulsant activity with improved protective index relative to standard of care sodium channel blockers. Epilepsia 63(3):697–708. 10.1111/epi.17149 35037706 10.1111/epi.17149 PMC9304232

[112] Kalume F, Yu FH, Westenbroek RE, Scheuer T, Catterall WA (2007) Reduced sodium current in Purkinje neurons from Nav1.1 mutant mice: implications for ataxia in severe myoclonic epilepsy in infancy. J Neurosci 27(41):11065–11074. 10.1523/JNEUROSCI.2162-07.2007 17928448 10.1523/JNEUROSCI.2162-07.2007 PMC6672849

[113] Kanellopoulos AH, Koenig J, Huang H, Pyrski M, Millet Q, Lolignier S, Morohashi T, Gossage SJ, Jay M, Linley JE, Baskozos G (2018) Mapping protein interactions of sodium channel Na_V_17 using epitope-tagged gene-targeted mice. EMBO J 37(3):427–445. 10.15252/embj.201796692 29335280 10.15252/embj.201796692 PMC5793798

[114] Kang YJ, Clement EM, Sumsky SL, Xiang Y, Park IH, Santaniello S et al (2020) The critical role of persistent sodium current in hippocampal gamma oscillations. Neuropharmacology 162:107787. 10.1016/j.neuropharm.2019.107787 31550457 10.1016/j.neuropharm.2019.107787 PMC6952064

[115] Katz E, Stoler O, Scheller A, Khrapunsky Y, Goebbels S, Kirchhoff F et al (2018) Role of sodium channel subtype in action potential generation by neocortical pyramidal neurons. Proc Natl Acad Sci USA 115(30):E7184–E7192. 10.1073/pnas.1720493115 29991598 10.1073/pnas.1720493115 PMC6065046

[116] Khaliq ZM, Bean BP (2010) Pacemaking in dopaminergic ventral tegmental area neurons: depolarizing drive from background and voltage-dependent sodium conductances. J Neurosci 30(21):7401–7413. 10.1523/JNEUROSCI.0143-10.2010 20505107 10.1523/JNEUROSCI.0143-10.2010 PMC2892804

[117] Kiehn J, Thomas D, Karle CA, Schöls W, Kübler W (1999) Inhibitory effects of the class III antiarrhythmic drug amiodarone on cloned HERG potassium channels. Arch Pharmacol 359(3):212–219. 10.1007/PL00005344 10.1007/pl00005344 10208308

[118] Kilpatrick ES, Forrest G, Brodie MJ (1996) Concentration–effect and concentration–toxicity relations with lamotrigine: a prospective study. Epilepsia 37(6):534–538. 10.1111/j.1528-1157.1996.tb00605.x 8641229 10.1111/j.1528-1157.1996.tb00605.x

[119] Kiss T (2008) Persistent Na-channels: origin and function. A review. Acta Biol Hung 59:1–12. 10.1556/ABiol.59.2008.Suppl.1 18652365 10.1556/ABiol.59.2008.Suppl.1

[120] Koizumi H, Smith JC (2008) Persistent Na+ and K+-dominated leak currents contribute to respiratory rhythm generation in the pre-Bötzinger complex in vitro. J Neurosci 28(7):1773–1785. 10.1523/JNEUROSCI.3916-07.2008 18272697 10.1523/JNEUROSCI.3916-07.2008 PMC6671552

[121] Kole MH (2011) First node of Ranvier facilitates high-frequency burst encoding. Neuron 71(4):671–682. 10.1016/j.neuron.2011.06.024 21867883 10.1016/j.neuron.2011.06.024

[122] Kononenko NI, Shao LR, Dudek FE (2004) Riluzole-sensitive slowly inactivating sodium current in rat suprachiasmatic nucleus neurons. J Neurophysiol 91(2):710–718. 10.1152/jn.00770.2003 14573554 10.1152/jn.00770.2003

[123] Kuo CC, Bean BP (1994) Slow binding of phenytoin to inactivated sodium channels in rat hippocampal neurons. Mol Pharmacol 46(4):716–725 7969051

[124] Kuo CC, Lu L (1997) Characterization of lamotrigine inhibition of Na+ channels in rat hippocampal neurones. Br J Pharmacol 121(6):1231–1238. 10.1038/sj.bjp.0701221 9249262 10.1038/sj.bjp.0701221 PMC1564785

[125] Kuo JJ, Siddique T, Fu R, Heckman CJ (2005) Increased persistent Na(+) current and its effect on excitability in motoneurones cultured from mutant SOD1 mice. J Physiol 563(Pt 3):843–854. 10.1113/jphysiol.2004.074138 15649979 10.1113/jphysiol.2004.074138 PMC1665614

[126] Kuwabara S, Misawa S (2008) Pharmacologic intervention in axonal excitability: in vivo assessment of nodal persistent sodium currents in human neuropathies. Curr Mol Pharmacol 1(1):61–67. 10.2174/1874467210801010061 20021424 10.2174/1874467210801010061

[127] Kuzmiski JB, Barr W, Zamponi GW, MacVicar BA (2005) Topiramate Inhibits the Initiation of Plateau Potentials in CA1 Neurons by Depressing R-type Calcium Channels. Epilepsia 46(4):481–489. 10.1111/j.0013-9580.2005.35304.x 15816941 10.1111/j.0013-9580.2005.35304.x

[128] Lamanauskas N, Nistri A (2008) Riluzole blocks persistent Na+ and Ca2+ currents and modulates release of glutamate via presynaptic NMDA receptors on neonatal rat hypoglossal motoneurons in vitro. Eur J Neurosci 27(10):2501–2514. 10.1111/j.1460-9568.2008.06211.x 18445055 10.1111/j.1460-9568.2008.06211.x

[129] Lamas JA, Romero M, Reboreda A, Sánchez E, Ribeiro SJ (2009) A riluzole- and valproate-sensitive persistent sodium current contributes to the resting membrane potential and increases the excitability of sympathetic neurones. Pflugers Arch 458(3):589–599. 10.1007/s00424-009-0648-0 19234716 10.1007/s00424-009-0648-0

[130] Lampl I, Schwindt P, Crill W (1998) Reduction of cortical pyramidal neuron excitability by the action of phenytoin on persistent Na+ current. J Pharmacol Exp Ther 284(1):228–237 9435183

[131] Lauxmann S, Sonnenberg L, Koch NA, Bosselmann C, Winter N, Schwarz N et al (2021) Therapeutic Potential of Sodium Channel Blockers as a Targeted Therapy Approach in KCNA1-Associated Episodic Ataxia and a Comprehensive Review of the Literature. Front Neurol 12. 10.3389/fneur.2021.703970 10.3389/fneur.2021.703970 PMC8459024 34566847

[132] Lee CH, Ruben PC (2008) Interaction between voltage-gated sodium channels and the neurotoxin, tetrodotoxin. Channels 2(6):407–412. 10.4161/chan.2.6.7429 19098433 10.4161/chan.2.6.7429

[133] Lenkey N, Karoly R, Lukacs P, Vizi ES, Sunesen M, Fodor L et al (2010) Classification of Drugs Based on Properties of Sodium Channel Inhibition: A Comparative Automated Patch-Clamp Study. PLoS One 5(12):e15568. 10.1371/journal.pone.0015568 21187965 10.1371/journal.pone.0015568 PMC3004914

[134] Leonard WA Jr (1958) The use of diphenylhydantoin (dilantin) sodium in the treatment of ventricular tachycardia. AMA Arch Intern Med 101(4):714–717. 10.1001/archinte.1958.00260160036005 13519913 10.1001/archinte.1958.00260160036005

[135] Leresche N, Parri HR, Erdemli G, Guyon A, Turner JP, Williams SR et al (1998) On the Action of the Anti-Absence Drug Ethosuximide in the Rat and Cat Thalamus. J Neurosci 18(13):4842–4853. 10.1523/jneurosci.18-13-04842.1998 9634550 10.1523/JNEUROSCI.18-13-04842.1998 PMC6792570

[136] Li Y, Gorassini MA, Bennett DJ (2004) Role of persistent sodium and calcium currents in motoneuron firing and spasticity in chronic spinal rats. J Neurophysiol 91(2):767–783. 10.1152/jn.00788.2003 14762149 10.1152/jn.00788.2003

[137] Liebeskind BJ, Hillis DM, Zakon HH (2011) Evolution of sodium channels predates the origin of nervous systems in animals. Proc Natl Acad Sci USA 108(22):9154–9159. 10.1073/pnas.1106363108 21576472 10.1073/pnas.1106363108 PMC3107268

[138] Lin Y-C, Lai Y-C, Lin T-H, Yang Y-C, Kuo C-C (2022) Selective stabilization of the intermediate inactivated Na+ channel by the new-generation anticonvulsant rufinamide. Biochem Pharmacol 197:114928. 10.1016/j.bcp.2022.114928 35063442 10.1016/j.bcp.2022.114928

[139] Lopez-Santiago LF, Yuan Y, Wagnon JL, Hull JM, Frasier CR, O’Malley HA et al (2017) Neuronal hyperexcitability in a mouse model of SCN8A epileptic encephalopathy. Proc Natl Acad Sci U S A 114(9):2383–2388. 10.1073/pnas.1616821114 28193882 10.1073/pnas.1616821114 PMC5338511

[140] Lunko O, Isaev D, Maximyuk O, Ivanchick G, Sydorenko V, Krishtal O et al (2014) Persistent sodium current properties in hippocampal CA1 pyramidal neurons of young and adult rats. Neurosci Lett 559:30–33. 10.1016/j.neulet.2013.11.035 24300033 10.1016/j.neulet.2013.11.035

[141] Ma JY, Catterall WA, Scheuer T (1997) Persistent sodium currents through brain sodium channels induced by G protein betagamma subunits. Neuron 19(2):443–452. 10.1016/s0896-6273(00)80952-6 9292732 10.1016/s0896-6273(00)80952-6

[142] Maltsev VA, Sabbah HN, Undrovinas AI (2001) Late Sodium Current is a Novel Target for Amiodarone: Studies in Failing Human Myocardium. J Mol Cell Cardiol 33(5):923–932. 10.1006/jmcc.2001.1355 11343415 10.1006/jmcc.2001.1355

[143] Mantegazza M, Yu FH, Powell AJ, Clare JJ, Catterall WA, Scheuer T (2005) Molecular determinants for modulation of persistent sodium current by G-protein betagamma subunits. J Neurosci 25(13):3341–3349. 10.1523/jneurosci.0104-05.2005 15800189 10.1523/JNEUROSCI.0104-05.2005 PMC6724911

[144] Markoula S, Teotonio R, Ratnaraj N, Duncan JS, Sander JW, Patsalos PN (2014) Lacosamide serum concentrations in adult patients with epilepsy: the influence of gender, age, dose, and concomitant antiepileptic drugs. Ther Drug Monit 36(4):494–498. 10.1097/ftd.0000000000000051 24562047 10.1097/FTD.0000000000000051

[145] Marson AG, Al-Kharusi AM, Alwaidh M, Appleton R, Baker GA, Chadwick DW et al (2007) The SANAD study of effectiveness of carbamazepine, gabapentin, lamotrigine, oxcarbazepine, or topiramate for treatment of partial epilepsy: an unblinded randomised controlled trial. Lancet 369(9566):1000–1015. 10.1016/s0140-6736(07)60460-7 17382827 10.1016/S0140-6736(07)60460-7 PMC2080688

[146] Marson AG, Al-Kharusi AM, Alwaidh M, Appleton R, Baker GA, Chadwick DW et al (2007) The SANAD study of effectiveness of valproate, lamotrigine, or topiramate for generalised and unclassifiable epilepsy: an unblinded randomised controlled trial. Lancet 369(9566):1016–1026. 10.1016/s0140-6736(07)60461-9 17382828 10.1016/S0140-6736(07)60461-9 PMC2039891

[147] Marson A, Burnside G, Appleton R, Smith D, Leach JP, Sills G et al (2021) The SANAD II study of the effectiveness and cost-effectiveness of levetiracetam, zonisamide, or lamotrigine for newly diagnosed focal epilepsy: an open-label, non-inferiority, multicentre, phase 4, randomised controlled trial. Lancet 397(10282):1363–1374. 10.1016/s0140-6736(21)00247-6 33838757 10.1016/S0140-6736(21)00247-6 PMC8047799

[148] Marson A, Burnside G, Appleton R, Smith D, Leach JP, Sills G et al (2021) The SANAD II study of the effectiveness and cost-effectiveness of valproate versus levetiracetam for newly diagnosed generalised and unclassifiable epilepsy: an open-label, non-inferiority, multicentre, phase 4, randomised controlled trial. Lancet 397(10282):1375–1386. 10.1016/S0140-6736(21)00246-4 33838758 10.1016/S0140-6736(21)00246-4 PMC8047813

[149] Martella G, De Persis C, Bonsi P, Natoli S, Cuomo D, Bernardi G et al (2005) Inhibition of Persistent Sodium Current Fraction and Voltage-gated L-type Calcium Current by Propofol in Cortical Neurons: Implications for Its Antiepileptic Activity. Epilepsia 46(5):624–635. 10.1111/j.1528-1167.2005.34904.x 15857426 10.1111/j.1528-1167.2005.34904.x

[150] Mason ER, Cummins TR (2020) Differential Inhibition of Human Nav1.2 Resurgent and Persistent Sodium Currents by Cannabidiol and GS967. Int J Mol Sci 21(7):2454. 10.3390/ijms21072454 32244818 10.3390/ijms21072454 PMC7177867

[151] Matsuki N, Quandt FN, Ten Eick RE, Yeh JZ (1984) Characterization of the block of sodium channels by phenytoin in mouse neuroblastoma cells. J Pharmacol Exp Ther 228(2):523–530 6319681

[152] Maurice N, Tkatch T, Meisler M, Sprunger LK, Surmeier DJ (2001) D1/D5 dopamine receptor activation differentially modulates rapidly inactivating and persistent sodium currents in prefrontal cortex pyramidal neurons. J Neurosci 21(7):2268–2277. 10.1523/jneurosci.21-07-02268.2001 11264302 10.1523/JNEUROSCI.21-07-02268.2001 PMC6762404

[153] May TW, Rambeck B, Jürgens U (2002) Serum Concentrations of Topiramate in Patients With Epilepsy: Influence of Dose, Age, and Comedication. Ther Drug Monit 2(3):366-374. 10.1097/00007691-200206000-00007 12021627 10.1097/00007691-200206000-00007

[154] Meng QT, Xia ZY, Liu J, Bayliss DA, Chen X (2011) Local anesthetic inhibits hyperpolarization-activated cationic currents. Mol Pharmacol 79(5):866–873. 10.1124/mol.110.070227 21303986 10.1124/mol.110.070227 PMC3082936

[155] Messing RO, Carpenter CL, Greenberg DA (1985) Mechanism of calcium channel inhibition by phenytoin: comparison with classical calcium channel antagonists. J Pharmacol Exp Ther 235(2):407–411 2414431

[156] Mittmann T, Alzheimer C (1998) Muscarinic inhibition of persistent Na+ current in rat neocortical pyramidal neurons. J Neurophysiol 79(3):1579–1582. 10.1152/jn.1998.79.3.1579 9497434 10.1152/jn.1998.79.3.1579

[157] Moutal A, François-Moutal L, Perez-Miller S, Cottier K, Chew LA, Yeon SK et al (2016) (S)-Lacosamide Binding to Collapsin Response Mediator Protein 2 (CRMP2) Regulates CaV2.2 Activity by Subverting Its Phosphorylation by Cdk5. Mol Neurobiol 53(3):1959–1976. 10.1007/s12035-015-9141-2 25846820 10.1007/s12035-015-9141-2

[158] Moutal A, Chew LA, Yang X, Wang Y, Yeon SK, Telemi E, Meroueh S, Park KD, Shrinivasan R, Gilbraith KB, Qu C, Xie JY, Patwardhan A, Vanderah TW, Khanna M, Porreca F, Khanna R (2016) (S)-lacosamide inhibition of CRMP2 phosphorylation reduces postoperative and neuropathic pain behaviors through distinct classes of sensory neurons identified by constellation pharmacology. Pain 157(7):1448–1463. 10.1097/j.pain.0000000000000555 26967696 10.1097/j.pain.0000000000000555 PMC4936788

[159] Mula M (2012) Topiramate and cognitive impairment: evidence and clinical implications. Ther Adv Drug Saf 3(6):279–289. 10.1177/2042098612455357 25083242 10.1177/2042098612455357 PMC4110841

[160] Müller P, Draguhn A, Egorov AV (2018) Persistent sodium current modulates axonal excitability in CA1 pyramidal neurons. J Neurochem 146(4):446–458. 10.1111/jnc.14479 29863287 10.1111/jnc.14479

[161] Muroi Y, Chanda B (2009) Local anesthetics disrupt energetic coupling between the voltage-sensing segments of a sodium channel. J Gen Physiol 133(1):1–15. 10.1085/jgp.200810103 19088384 10.1085/jgp.200810103 PMC2606943

[162] Murphy R, Alle H, Geiger JRP, Storm JF (2024) Estimation of persistent sodium-current density in rat hippocampal mossy fibre boutons: Correction of space-clamp errors. J Physiol 602(8):1703–1732. 10.1113/JP284657 38594842 10.1113/JP284657

[163] Nakamura M, Cho JH, Shin H, Jang IS (2019) Effects of cenobamate (YKP3089), a newly developed anti-epileptic drug, on voltage-gated sodium channels in rat hippocampal CA3 neurons. Eur J Pharmacol 855:175–182. 10.1016/j.ejphar.2019.05.007 31063770 10.1016/j.ejphar.2019.05.007

[164] Narahashi T (2001) Pharmacology of tetrodotoxin. J Toxicol Toxin Rev 20(1):67–84. 10.1081/TXR-100102537

[165] Niespodziany I, Klitgaard H, Margineanu DG (2004) Is the persistent sodium current a specific target of anti-absence drugs? NeuroReport 15(6):1049–1052. 10.1097/00001756-200404290-00023 15076732 10.1097/00001756-200404290-00023

[166] Niespodziany I, Leclère N, Vandenplas C, Foerch P, Wolff C (2013) Comparative study of lacosamide and classical sodium channel blocking antiepileptic drugs on sodium channel slow inactivation. J Neurosci Res 91(3):436–443. 10.1002/jnr.23136 23239147 10.1002/jnr.23136

[167] Park YY, Johnston D, Gray R (2013) Slowly inactivating component of Na+ current in peri-somatic region of hippocampal CA1 pyramidal neurons. J Neurophysiol 109(5):1378–1390. 10.1152/jn.00435.2012 23236005 10.1152/jn.00435.2012 PMC3602831

[168] Park SB, Kiernan MC, Vucic S (2017) Axonal Excitability in Amyotrophic Lateral Sclerosis : Axonal Excitability in ALS. Neurotherapeutics 14(1):78–90. 10.1007/s13311-016-0492-9 27878516 10.1007/s13311-016-0492-9 PMC5233634

[169] Patel R, Dickenson AH (2016) Mechanisms of the gabapentinoids and α 2 δ-1 calcium channel subunit in neuropathic pain. Pharmacol Res Perspect 4(2):e00205. 10.1002/prp2.205 27069626 10.1002/prp2.205 PMC4804325

[170] Patel RR, Barbosa C, Brustovetsky T, Brustovetsky N, Cummins TR (2016) Aberrant epilepsy-associated mutant Nav1.6 sodium channel activity can be targeted with cannabidiol. Brain 139(Pt 8):2164–2181. 10.1093/brain/aww129 27267376 10.1093/brain/aww129 PMC4958898

[171] Patlak JB, Ortiz M (1986) Two modes of gating during late Na+ channel currents in frog sartorius muscle. J Gen Physiol 87(2):305–326. 10.1085/jgp.87.2.305 2419486 10.1085/jgp.87.2.305 PMC2217600

[172] Perucca E (2007) Treatment of epilepsy in developing countries. BMJ 334(7605):1175–1176. 10.1136/bmj.39065.460208.80 17556434 10.1136/bmj.39065.460208.80 PMC1889934

[173] Pieri M, Carunchio I, Curcio L, Mercuri NB, Zona C (2009) Increased persistent sodium current determines cortical hyperexcitability in a genetic model of amyotrophic lateral sclerosis. Exp Neurol 215(2):368–379. 10.1016/j.expneurol.2008.11.002 19071115 10.1016/j.expneurol.2008.11.002

[174] Prakriya M, Mennerick S (2000) Selective Depression of Low-Release Probability Excitatory Synapses by Sodium Channel Blockers. Neuron 26(3):671–682. 10.1016/S0896-6273(00)81203-9 10896162 10.1016/s0896-6273(00)81203-9

[175] Ptak K, Zummo GG, Alheid GF, Tkatch T, Surmeier DJ, McCrimmon DR (2005) Sodium Currents in Medullary Neurons Isolated from the Pre-Bötzinger Complex Region. J Neurosci 25(21):5159–5170. 10.1523/jneurosci.4238-04.2005 15917456 10.1523/JNEUROSCI.4238-04.2005 PMC6724824

[176] Quandt FN (1988) Modification of slow inactivation of single sodium channels by phenytoin in neuroblastoma cells. Mol Pharmacol 34(4):557–565 2845252

[177] Rayner‐Hartley E, Sedlak T (2016) Ranolazine: A Contemporary Review. J Am Heart Assoc 5(3):e003196. 10.1161/JAHA.116.003196 10.1161/JAHA.116.003196 PMC4943285 26979079

[178] Ren S-c, Chen P-z, Jiang H-h, Mi Z, Xu F, Hu B et al (2014) Persistent sodium currents contribute to Aβ1-42-induced hyperexcitation of hippocampal CA1 pyramidal neurons. Neurosci Lett 580:62–67. 10.1016/j.neulet.2014.07.050 25102326 10.1016/j.neulet.2014.07.050

[179] Richens A (1979) Clinical pharmacokinetics of phenytoin. Clin Pharmacokinet 4(3):153–169. 10.2165/00003088-197904030-00001 383353 10.2165/00003088-197904030-00001

[180] Riva E, Gerna M, Neyroz P, Urso R, Bartosek I, Guaitani A (1982) Pharmacokinetics of Amiodarone in Rats. J Cardiovasc Pharmacol 4(2):270–275. 10.1097/00005344-198203000-00016 6175811 10.1097/00005344-198203000-00016

[181] Rojas E, Rudy B (1976) Destruction of the sodium conductance inactivation by a specific protease in perfused nerve fibres from Loligo. J Physiol 262(2):501–531. 10.1113/jphysiol.1976.sp011608 994046 10.1113/jphysiol.1976.sp011608 PMC1307656

[182] Royeck M, Horstmann M-T, Remy S, Reitze M, Yaari Y, Beck H (2008) Role of Axonal NaV1.6 Sodium Channels in Action Potential Initiation of CA1 Pyramidal Neurons. J Neurophysiol 100(4):2361–2380. 10.1152/jn.90332.2008 18650312 10.1152/jn.90332.2008

[183] Royeck M, Kelly T, Opitz T, Otte DM, Rennhack A, Woitecki A et al (2015) Downregulation of Spermine Augments Dendritic Persistent Sodium Currents and Synaptic Integration after Status Epilepticus. J Neurosci 35(46):15240–15253. 10.1523/jneurosci.0493-15.2015 26586813 10.1523/JNEUROSCI.0493-15.2015 PMC6605494

[184] Rudy B (1978) Slow inactivation of the sodium conductance in squid giant axons. Pronase resistance. J Physiol 283:1–21. 10.1113/jphysiol.1978.sp012485 722569 10.1113/jphysiol.1978.sp012485 PMC1282762

[185] Rühlmann AH, Körner J, Hausmann R, Bebrivenski N, Neuhof C, Detro-Dassen S et al (2020) Uncoupling sodium channel dimers restores the phenotype of a pain-linked Nav1.7 channel mutation. Br J Pharmacol 177:4481–4496. 10.1111/bph.15196 32663327 10.1111/bph.15196 PMC7484505

[186] Rush AM, Dib-Hajj SD, Waxman SG (2005) Electrophysiological properties of two axonal sodium channels, Nav1.2 and Nav1.6, expressed in mouse spinal sensory neurones. J Physiol 564(Pt 3):803–815. 10.1113/jphysiol.2005.083089 15760941 10.1113/jphysiol.2005.083089 PMC1464456

[187] Russell JL, Spiller HA, Baker DD (2015) Markedly Elevated Carbamazepine-10,11-epoxide/Carbamazepine Ratio in a Fatal Carbamazepine Ingestion. Case Report Med 2015:369707. 10.1155/2015/369707 10.1155/2015/369707 PMC4621337 26550016

[188] Russo EB, Burnett A, Hall B, Parker KK (2005) Agonistic Properties of Cannabidiol at 5-HT1a Receptors. Neurochem Res 30(8):1037–1043. 10.1007/s11064-005-6978-1 16258853 10.1007/s11064-005-6978-1

[189] Sahinovic MM, Struys MMRF, Absalom AR (2018) Clinical Pharmacokinetics and Pharmacodynamics of Propofol. Clin Pharmacokinet 57(12):1539–1558. 10.1007/s40262-018-0672-3 30019172 10.1007/s40262-018-0672-3 PMC6267518

[190] Schauf CL (1987) Zonisamide enhances slow sodium inactivation in Myxicola. Brain Res 413(1):185–188. 10.1016/0006-8993(87)90168-5 2439177 10.1016/0006-8993(87)90168-5

[191] Schik G, Wedegaertner FR, Liersch J, Hoy L, Emrich HM, Schneider U (2005) Oxcarbazepine versus carbamazepine in the treatment of alcohol withdrawal. Addict Biol 10(3):283–238. 10.1080/13556210500224015 16109591 10.1080/13556210500224015

[192] Segal MM, Douglas AF (1997) Late sodium channel openings underlying epileptiform activity are preferentially diminished by the anticonvulsant phenytoin. J Neurophysiol 77(6):3021–2034. 10.1152/jn.1997.77.6.3021 9212254 10.1152/jn.1997.77.6.3021

[193] Shank RP, Gardocki JF, Vaught JL, Davis CB, Schupsky JJ, Raffa RB et al (1994) Topiramate: preclinical evaluation of structurally novel anticonvulsant. Epilepsia 35(2):450–460. 10.1111/j.1528-1157.1994.tb02459.x 8156972 10.1111/j.1528-1157.1994.tb02459.x

[194] Sharma R, Nakamura M, Neupane C, Jeon BH, Shin H, Melnick SM et al (2020) Positive allosteric modulation of GABAA receptors by a novel antiepileptic drug cenobamate. Eur J Pharmacol 879:173117. 10.1016/j.ejphar.2020.173117 32325146 10.1016/j.ejphar.2020.173117

[195] Sheets PL, Heers C, Stoehr T, Cummins TR (2008) Differential block of sensory neuronal voltage-gated sodium channels by lacosamide [(2R)-2-(acetylamino)-N-benzyl-3-methoxypropanamide], lidocaine, and carbamazepine. J Pharmacol Exp Ther 326(1):89–99. 10.1124/jpet.107.133413 18378801 10.1124/jpet.107.133413

[196] Sheroziya MG, Egorov AV (2010) Effects of extracellular calcium on the volley activity of entorhinal cortex neurons in neonatal rats: computer simulation. Neurosci Behav Physiol 40(1):1–4. 10.1007/s11055-009-9229-0 20012490 10.1007/s11055-009-9229-0

[197] Sheroziya MG, Halbach OV, Unsicker K, Egorov AV (2009) Spontaneous bursting activity in the developing entorhinal cortex. J Neurosci 29(39):12131–12144. 10.1523/JNEUROSCI.1333-09.2009 19793971 10.1523/JNEUROSCI.1333-09.2009 PMC6666150

[198] Shi QQ, Sun X, Fang H (2014) A mechanism study on propofol’s action on middle latency auditory evoked potential by neurons in ventral partition of medial geniculate body in rats. Eur Rev Med Pharmacol Sci 18(13):1859–1868 25010614

[199] Shibuya K, Misawa S, Kimura H, Noto Y-i, Sekiguchi Y, Iwai Y et al (2016) Increased motor axonal persistent sodium currents predict rapid functional declines in amyotrophic lateral sclerosis. Neurol Clin Neurosci 4(3):108–111. 10.1111/ncn3.12044

[200] Silver KS, Du Y, Nomura Y, Oliveira EE, Salgado VL, Zhorov BS et al (2014) Voltage-Gated Sodium Channels as Insecticide Targets. Adv In Insect Phys 46:389–433. 10.1016/B978-0-12-417010-0.00005-7 29928068 10.1016/B978-0-12-417010-0.00005-7 PMC6005695

[201] Sipilä ST, Huttu K, Voipio J, Kaila K (2006) Intrinsic bursting of immature CA3 pyramidal neurons and consequent giant depolarizing potentials are driven by a persistent Na+ current and terminated by a slow Ca2+ -activated K+ current. Eur J Neurosci 23(9):2330–2338. 10.1111/j.1460-9568.2006.04757.x 16706841 10.1111/j.1460-9568.2006.04757.x

[202] Soar J, Böttiger BW, Carli P, Couper K, Deakin CD, Djärv T et al (2021) European Resuscitation Council Guidelines 2021: Adult advanced life support. Resuscitation 161:115–151. 10.1016/j.resuscitation.2021.02.010 33773825 10.1016/j.resuscitation.2021.02.010

[203] Soares-da-Silva P, Pires N, Bonifácio MJ, Loureiro AI, Palma N, Wright LC (2015) Eslicarbazepine acetate for the treatment of focal epilepsy: an update on its proposed mechanisms of action. Pharmacol Res Perspect 3(2):e00124. 10.1002/prp2.124 26038700 10.1002/prp2.124 PMC4448990

[204] Southam E, Kirkby D, Higgins GA, Hagan RM (1998) Lamotrigine inhibits monoamine uptake in vitro and modulates 5-hydroxytryptamine uptake in rats. Eur J Pharmacol 358(1):19–24. 10.1016/s0014-2999(98)00580-9 9809864 10.1016/s0014-2999(98)00580-9

[205] Spadoni F, Hainsworth AH, Mercuri NB, Caputi L, Martella G, Lavaroni F et al (2002) Lamotrigine derivatives and riluzole inhibit INa, P in cortical neurons. NeuroReport 13(9):1167–1170. 10.1097/00001756-200207020-00019 12151762 10.1097/00001756-200207020-00019

[206] Stafstrom CE (2007) Persistent sodium current and its role in epilepsy. Epilepsy Curr 7(1):15–22. 10.1111/j.1535-7511.2007.00156.x 17304346 10.1111/j.1535-7511.2007.00156.x PMC1797888

[207] Stafstrom CE, Schwindt PC, Chubb MC, Crill WE (1985) Properties of persistent sodium conductance and calcium conductance of layer V neurons from cat sensorimotor cortex in vitro. J Neurophysiol 53(1):153–170. 10.1152/jn.1985.53.1.153 2579215 10.1152/jn.1985.53.1.153

[208] Stefani A, Pisani A, De Murtas M, Mercuri NB, Marciani MG, Calabresi P (1995) Action of GP 47779, the Active Metabolite of Oxcarbazepine, on the Corticostriatal System. II. Modulation of High-Voltage-Activated Calcium Currents. Epilepsia 36(10):997–1002. 10.1111/j.1528-1157.1995.tb00958.x 7555964 10.1111/j.1528-1157.1995.tb00958.x

[209] Stefani A, Spadoni F, Siniscalchi A, Bernardi G (1996) Lamotrigine inhibits Ca2+ currents in cortical neurons: functional implications. Eur J Pharmacol 307(1):113–116. 10.1016/0014-2999(96)00265-8 8831112 10.1016/0014-2999(96)00265-8

[210] Storm J, Vervaeke K, Hu H, Graham L. Functions of the Persistent Na+ Current in Cortical Neurons Revealed by Dynamic Clamp. Dynamic-Clamp. New York, NY: Springer; 2009. p. 165–197 10.1007/978-0-387-89279-5_8

[211] Stuart G (1999) Voltage–activated sodium channels amplify inhibition in neocortical pyramidal neurons. Nat Neurosci 2(2):144–150. 10.1038/5698 10195198 10.1038/5698

[212] Stuart G, Sakmann B (1995) Amplification of EPSPs by axosomatic sodium channels in neocortical pyramidal neurons. Neuron 15(5):1065–1076. 10.1016/0896-6273(95)90095-0 7576650 10.1016/0896-6273(95)90095-0

[213] Stys PK, Sontheimer H, Ransom BR, Waxman SG (1993) Noninactivating, tetrodotoxin-sensitive Na+ conductance in rat optic nerve axons. Proc Natl Acad Sci U S A 90(15):6976–6980. 10.1073/pnas.90.15.6976 8394004 10.1073/pnas.90.15.6976 PMC47058

[214] Su H, Alroy G, Kirson ED, Yaari Y (2001) Extracellular calcium modulates persistent sodium current-dependent burst-firing in hippocampal pyramidal neurons. J Neurosci 21(12):4173–4182. 10.1523/jneurosci.21-12-04173.2001 11404402 10.1523/JNEUROSCI.21-12-04173.2001 PMC6762760

[215] Sun G-c, Werkman TR, Battefeld A, Clare JJ, Wadman WJ (2007) Carbamazepine and Topiramate Modulation of Transient and Persistent Sodium Currents Studied in HEK293 Cells Expressing the Nav1.3 α–Subunit. Epilepsia 48(4):774–782. 10.1111/j.1528-1167.2007.01001.x 17381447 10.1111/j.1528-1167.2007.01001.x

[216] Taddese A, Bean BP (2002) Subthreshold sodium current from rapidly inactivating sodium channels drives spontaneous firing of tuberomammillary neurons. Neuron 33(4):587–600. 10.1016/s0896-6273(02)00574-3 11856532 10.1016/s0896-6273(02)00574-3

[217] Taverna S, Mantegazza M, Franceschetti S, Avanzini G (1998) Valproate selectively reduces the persistent fraction of Na+ current in neocortical neurons. Epilepsy Res 32(1):304–308. 10.1016/S0920-1211(98)00060-6 9761329 10.1016/s0920-1211(98)00060-6

[218] Taverna S, Sancini G, Mantegazza M, Franceschetti S, Avanzini G (1999) Inhibition of transient and persistent Na+ current fractions by the new anticonvulsant topiramate. J Pharmacol Exp Ther 288(3):960–968. 10027832

[219] Taylor JC, Brauer S, Espir ML (1981) Long-term treatment of trigeminal neuralgia with carbamazepine. Postgrad Med J 57(663):16–18. 10.1136/pgmj.57.663.16 7279817 10.1136/pgmj.57.663.16 PMC2424760

[220] Tazerart S, Vinay L, Brocard F (2008) The persistent sodium current generates pacemaker activities in the central pattern generator for locomotion and regulates the locomotor rhythm. J Neurosci 28(34):8577–8589. 10.1523/JNEUROSCI.1437-08.2008 18716217 10.1523/JNEUROSCI.1437-08.2008 PMC6671046

[221] Theiss RD, Kuo JJ, Heckman CJ (2007) Persistent inward currents in rat ventral horn neurones. J Physiol 580(2):507–522. 10.1113/jphysiol.2006.124123 17289788 10.1113/jphysiol.2006.124123 PMC2075552

[222] Thomas A, Baillie GL, Phillips AM, Razdan RK, Ross RA, Pertwee RG (2007) Cannabidiol displays unexpectedly high potency as an antagonist of CB1 and CB2 receptor agonists in vitro. Br J Pharmacol 150(5):613–623. 10.1038/sj.bjp.0707133 17245363 10.1038/sj.bjp.0707133 PMC2189767

[223] Tomson T, Battino D, Bonizzoni E, Craig J, Lindhout D, Perucca E et al (2015) Dose-dependent teratogenicity of valproate in mono- and polytherapy: an observational study. Neurology 85(10):866–872. 10.1212/wnl.0000000000001772 26085607 10.1212/WNL.0000000000001772

[224] Tomson T, Battino D, Bonizzoni E, Craig J, Lindhout D, Perucca E et al (2018) Comparative risk of major congenital malformations with eight different antiepileptic drugs: a prospective cohort study of the EURAP registry. Lancet Neurol 17(6):530–538. 10.1016/S1474-4422(18)30107-8 29680205 10.1016/S1474-4422(18)30107-8

[225] Trapp BD, Stys PK (2009) Virtual hypoxia and chronic necrosis of demyelinated axons in multiple sclerosis. Lancet Neurol 8(3):280–91. 10.1016/s1474-4422(09)70043-2 19233038 10.1016/S1474-4422(09)70043-2

[226] Uebachs M, Opitz T, Royeck M, Dickhof G, Horstmann M-T, Isom LL et al (2010) Efficacy Loss of the Anticonvulsant Carbamazepine in Mice Lacking Sodium Channel β Subunits via Paradoxical Effects on Persistent Sodium Currents. J Neurosci 30(25):8489–8501. 10.1523/jneurosci.1534-10.2010 20573896 10.1523/JNEUROSCI.1534-10.2010 PMC6634624

[227] Uebachs M, Albus C, Opitz T, Isom L, Niespodziany I, Wolff C et al (2012) Loss of β1 accessory Na+ channel subunits causes failure of carbamazepine, but not of lacosamide, in blocking high-frequency firing via differential effects on persistent Na+ currents. Epilepsia 53(11):1959–1967. 10.1111/j.1528-1167.2012.03675.x 23016711 10.1111/j.1528-1167.2012.03675.x

[228] Urbani A, Belluzzi O (2000) Riluzole inhibits the persistent sodium current in mammalian CNS neurons. Eur J Neurosci 12(10):3567–3574. 10.1046/j.1460-9568.2000.00242.x 11029626 10.1046/j.1460-9568.2000.00242.x

[229] Uteshev V, Stevens DR, Haas HL (1995) A persistent sodium current in acutely isolated histaminergic neurons from rat hypothalamus. Neuroscience 66(1):143–149. 10.1016/0306-4522(94)00593-t 7637864 10.1016/0306-4522(94)00593-t

[230] Van den Berg RJ, Kok P, Voskuyl RA (1993) Valproate and sodium currents in cultured hippocampal neurons. Exp Brain Res 93(2):279–287. 10.1007/BF00228395 8387930 10.1007/BF00228395

[231] van Drongelen W, Koch H, Elsen FP, Lee HC, Mrejeru A, Doren E et al (2006) Role of Persistent Sodium Current in Bursting Activity of Mouse Neocortical Networks In Vitro. J Neurophysiol 96(5):2564–2577. 10.1152/jn.00446.2006 16870839 10.1152/jn.00446.2006

[232] Veeramah KR, O’Brien JE, Meisler MH, Cheng X, Dib-Hajj SD, Waxman SG et al (2012) De novo pathogenic SCN8A mutation identified by whole-genome sequencing of a family quartet affected by infantile epileptic encephalopathy and SUDEP. Am J Hum Genet 90(3):502–510. 10.1016/j.ajhg.2012.01.006 22365152 10.1016/j.ajhg.2012.01.006 PMC3309181

[233] Vera J, Alcayaga J, Sanhueza M (2017) Competition between Persistent Na(+) and Muscarine-Sensitive K(+) Currents Shapes Perithreshold Resonance and Spike Tuning in CA1 Pyramidal Neurons. Front Cell Neurosci 11:61. 10.3389/fncel.2017.00061 28337126 10.3389/fncel.2017.00061 PMC5340745

[234] Vervaeke K, Hu H, Graham LJ, Storm JF (2006) Contrasting effects of the persistent Na+ current on neuronal excitability and spike timing. Neuron 49(2):257–270. 10.1016/j.neuron.2005.12.022 16423699 10.1016/j.neuron.2005.12.022

[235] Vreugdenhil M, Hoogland G, van Veelen CW, Wadman WJ (2004) Persistent sodium current in subicular neurons isolated from patients with temporal lobe epilepsy. Eur J Neurosci 19(10):2769–2778. 10.1111/j.1460-9568.2004.03400.x 15147310 10.1111/j.1460-9568.2004.03400.x

[236] Wang SJ, Sihra TS, Gean PW (2001) Lamotrigine inhibition of glutamate release from isolated cerebrocortical nerve terminals (synaptosomes) by suppression of voltage-activated calcium channel activity. NeuroReport 12(10):2255–2258. 10.1097/00001756-200107200-00042 11447345 10.1097/00001756-200107200-00042

[237] Wang J-F, Sun X, Chen B, Young LT (2002) Lamotrigine Increases Gene Expression of GABA-A Receptor β3 Subunit in Primary Cultured Rat Hippocampus Cells. Neuropsychopharmacology 26(4):415–421. 10.1016/S0893-133X(01)00385-2 11927166 10.1016/S0893-133X(01)00385-2

[238] Wasserstrom JA, Salata JJ (1988) Basis for tetrodotoxin and lidocaine effects on action potentials in dog ventricular myocytes. Am J Physiol Heart Circ Physiol 254(6):H1157–H1166. 10.1152/ajpheart.1988.254.6.H1157 10.1152/ajpheart.1988.254.6.H1157 2454585

[239] Wengert ER, Patel MK (2020) The Role of the Persistent Sodium Current in Epilepsy. Epilepsy Curr 21(1):40–47. 10.1177/1535759720973978 33236643 10.1177/1535759720973978 PMC7863310

[240] Wengert ER, Saga AU, Panchal PS, Barker BS, Patel MK (2019) Prax330 reduces persistent and resurgent sodium channel currents and neuronal hyperexcitability of subiculum neurons in a mouse model of SCN8A epileptic encephalopathy. Neuropharmacology 158:107699–107699. 10.1016/j.neuropharm.2019.107699 31278928 10.1016/j.neuropharm.2019.107699 PMC6745260

[241] White HS, Brown SD, Woodhead JH, Skeen GA, Wolf HH (1997) Topiramate enhances GABA-mediated chloride flux and GABA-evoked chloride currents in murine brain neurons and increases seizure threshold. Epilepsy Res 28(3):167–179. 10.1016/S0920-1211(97)00045-4 9332882 10.1016/s0920-1211(97)00045-4

[242] Wu N, Enomoto A, Tanaka S, Hsiao CF, Nykamp DQ, Izhikevich E et al (2005) Persistent sodium currents in mesencephalic v neurons participate in burst generation and control of membrane excitability. J Neurophysiol 93(5):2710–2722. 10.1152/jn.00636.2004 15625100 10.1152/jn.00636.2004

[243] Wu L, Rajamani S, Shryock JC, Li H, Ruskin J, Antzelevitch C et al (2007) Augmentation of late sodium current unmasks the proarrhythmic effects of amiodarone. Cardiovasc Res 77(3):481–488. 10.1093/cvr/cvm06 18006430 10.1093/cvr/cvm069 PMC2365898

[244] Wu S-N, Chen B-S, Hsu T-I, Peng H, Wu Y-H, Lo Y-C (2009) Analytical studies of rapidly inactivating and noninactivating sodium currents in differentiated NG108–15 neuronal cells. J Theor Biol 259(4):828–836. 10.1016/j.jtbi.2009.05.003 19446569 10.1016/j.jtbi.2009.05.003

[245] Wu P-M, Cho H-Y, Chiang C-W, Chuang T-H, Wu S-N, Tu Y-F (2022) Characterization in Inhibitory Effectiveness of Carbamazepine in Voltage-Gated Na+ and Erg-Mediated K+ Currents in a Mouse Neural Crest-Derived (Neuro-2a) Cell Line. Int J Mol Sci 23(14):7892. 10.3390/ijms23147892 35887240 10.3390/ijms23147892 PMC9321339

[246] Xie R-G, Zheng D-W, Xing J, Zhang X-J, Song Y, Xie Y-B et al (2011) Blockade of Persistent Sodium Currents Contributes to the Riluzole-Induced Inhibition of Spontaneous Activity and Oscillations in Injured DRG Neurons. PLoS ONE 6:e18681. 10.1371/journal.pone.0018681 21541342 10.1371/journal.pone.0018681 PMC3081829

[247] Yadav R, Schrem E, Yadav V, Jayarangaiah A, Das S, Theetha Kariyanna P (2021) Lacosamide-Related Arrhythmias: A Systematic Analysis and Review of the Literature. Cureus 13(12):e20736. 10.7759/cureus.20736 35111429 10.7759/cureus.20736 PMC8790938

[248] Yamada-Hanff J, Bean BP (2013) Persistent sodium current drives conditional pacemaking in CA1 pyramidal neurons under muscarinic stimulation. J Neurosci 33(38):15011–15021. 10.1523/jneurosci.0577-13.2013 24048831 10.1523/JNEUROSCI.0577-13.2013 PMC3776055

[249] Yamada-Hanff J, Bean BP (2015) Activation of Ih and TTX-sensitive sodium current at subthreshold voltages during CA1 pyramidal neuron firing. J Neurophysiol 114(4):2376–2389. 10.1152/jn.00489.2015 26289465 10.1152/jn.00489.2015 PMC4620139

[250] Yang R-H, Wang W-T, Chen J-Y, Xie R-G, Hu S-J (2009) Gabapentin selectively reduces persistent sodium current in injured type-A dorsal root ganglion neurons. Pain 143(1):48–55. 10.1016/j.pain.2009.01.020 19269740 10.1016/j.pain.2009.01.020

[251] Yue C, Remy S, Su H, Beck H, Yaari Y (2005) Proximal persistent Na+ channels drive spike afterdepolarizations and associated bursting in adult CA1 pyramidal cells. J Neurosci 25(42):9704–9720. 10.1523/jneurosci.1621-05.2005 16237175 10.1523/JNEUROSCI.1621-05.2005 PMC6725731

[252] Zeiler FA, Zeiler KJ, Kazina CJ, Teitelbaum J, Gillman LM, West M (2015) Lidocaine for status epilepticus in adults. Seizure 31:41–48. 10.1016/j.seizure.2015.07.003 26362376 10.1016/j.seizure.2015.07.003

[253] Zeng Z, Hill-Yardin EL, Williams D, O’Brien T, Serelis A, French CR (2016) Effect of phenytoin on sodium conductances in rat hippocampal CA1 pyramidal neurons. J Neurophysiol 116(4):1924–1936. 10.1152/jn.01060.2015 27489371 10.1152/jn.01060.2015 PMC5144711

[254] Zhang H-XB, Bean BP (2021) Cannabidiol Inhibition of Murine Primary Nociceptors: Tight Binding to Slow Inactivated States of Nav18 Channels. J Neurosci 41(30):6371–6387. 10.1523/jneurosci.3216-20.2021 34131037 10.1523/JNEUROSCI.3216-20.2021 PMC8318087

[255] Zhang HB, Heckman L, Niday Z, Jo S, Fujita A, Shim J, et al (2022) Cannabidiol activates neuronal Kv7 channels. Elife 11 10.7554/eLife.73246 10.7554/eLife.73246 PMC8856652 35179483

