# Correction to: Persistent sodium currents in neurons: potential mechanisms and pharmacological blockers

**DOI:** 10.1007/s00424-024-02995-0

**Published:** 2024-07-24

**Authors:** Peter Müller, Andreas Draguhn, Alexei V. Egorov

**Affiliations:** 1 Department Neurology and Epileptology, Hertie Institute for Clinical Brain Research, University of Tuebingen, Hoppe-Seyler-Straße 3, 72076 Tübingen, Germany; 2 Institute for Physiology and Pathophysiology, Medical Faculty, Heidelberg University, Im Neuenheimer Feld 326, 69120 Heidelberg, Germany

**Correction to: Pfügers Archiv - European Journal of Physiology (2024)**

10.1007/s00424-024-02980-7

The original version of this article, published on 05 July 2024, unfortunately contained a mistake.

In this article, the references have been incorrectly assigned. The correct assignment of references can be found on the following pages. Pages: 1-4, 6-7, 9-12 and 14-21.

The publisher apologizes for the error.

The original article has been corrected.

